# Association between 23 drugs and inflammatory bowel disease: a two-sample Mendelian randomization study

**DOI:** 10.3389/fmed.2024.1371362

**Published:** 2024-05-21

**Authors:** Lei He, Tuo Deng, Yurong Huang, Wangliu Yang, Jie Yang, Gengqing Song

**Affiliations:** ^1^Department of Gastroenterology, The Affiliated Hospital of Guizhou Medical University, Guiyang, China; ^2^Department of Gastroenterology, Liupanshui People's Hospital, Liupanshui, Guizhou, China; ^3^Department of Gastroenterology and Hepatology, Metrohealth Medical Center, Case Western Reserve University, Cleveland, OH, United States

**Keywords:** inflammatory bowel disease, ulcerative colitis, Crohn’s disease, Mendelian randomization, 23 drugs

## Abstract

**Background:**

Inflammatory bowel disease (IBD) is a group of diseases characterized by chronic and recurrent inflammation of the gastrointestinal tract. The etiology of IBD remains multifaceted and poorly understood, resulting in limited treatment options that primarily target disease induction and remission maintenance. Thus, the exploration of novel therapeutic options for IBD among existing medications is advantageous. Mendelian randomization analysis (MR) serves as a valuable tool in investigating the relationship between drugs and diseases. In this study, MR analysis was employed to investigate the potential causal relationship between 23 approved drugs for the treatment of various diseases and IBD.

**Method:**

We performed a two-sample MR analysis using publicly available genome-wide association study (GWAS) statistics. The inverse variance weighting (IVW) method was used as the main analysis method, supplemented by the remaining four methods (weighted median, MR Egger regression, simple and weighted models), and Meta-analysis was performed to expand the sample size to obtain a more reliable composite causal effect. Finally, Cochran’s Q statistic and the MR-Egger test for directed pleiotropy were applied to determine whether significant heterogeneity or directed pleiotropy existed.

**Results:**

In the main MR analysis (IVW), drugs with a negative causal association with the risk of IBD were immunosuppressant {OR (95% CI) = 0.7389 [0.6311–0.8651], *p* = 0.0046} and diabetes drugs {OR (95% CI) = 0.9266 [0.8876–0.9674], *p* = 0.0058}. A positive causal association with the risk of IBD was found for salicylic acid and derivatives {OR (95% CI) = 1.2737 [1.0778–1.5053], *p* = 0.0345}. Negative causal associations with UC risk were identified for immunosuppressants {OR (95% CI) = 0.6660 [0.5133–0.8640], *p* = 0.0169} and diabetes medications {OR (95% CI) = 0.9020 [0.8508–0.9551], *p* = 0.0046}; positive causal associations with UC risk were found for β-receptor blockers {OR (95% CI) = 1.1893 [1.0823–1.3070], *p* = 0.0046}. A negative causal association with the risk of CD was found for immunosuppressants {OR (95% CI) = 0.6957 [0.5803–0.8341], *p* = 0.0023}. There was no statistically significant association between the remaining 19 drugs and IBD and subtypes.

**Conclusion:**

This MR study provides evidence suggesting that immunosuppressants have a mitigating effect on the risk of IBD and demonstrate consistent efficacy in subtypes of ulcerative colitis (UC) and Crohn’s disease (CD). Additionally, diabetes medications show potential in reducing the risk of IBD, particularly in cases of UC, while β-blockers may elevate the risk of UC. Conversely, salicylic acid and its derivatives may increase the risk of IBD, although this effect is not consistently observed in the subtypes of the disease. These findings offer new insights into the prevention and management of IBD.

## Introduction

Inflammatory bowel disease, a chronic inflammatory disorder of the gastrointestinal tract marked by dysregulation of the intestinal immune response (1), encompasses two primary conditions: ulcerative colitis and Crohn’s disease (2). The prevalence of IBD has surged worldwide with the advent of global industrialization, affecting not only developed nations like North America, Europe, Australia, and New Zealand, but also emerging regions in Asia and South America, as well as developing countries such as Brazil, South Korea, and China (3–5). Over the past decade, there has been a notable increase in the number of cases of Inflammatory Bowel Disease in China, rising from 1.72 cases per 100,000 to 3.35 cases per 100,000 in males and from 1.20 cases per 100,000 to 2.65 cases per 100,000 in females (5). The etiology of IBD is complex and not fully understood, resulting in limited treatment options. This lack of effective treatment options places a significant strain on public healthcare systems and presents a global public health challenge (6). Traditional treatment for inflammatory bowel disease typically involves the use of medications like aminosalicylates and corticosteroids to manage symptoms, as well as other interventions such as surgical excision when deemed necessary (7). However, certain individuals with inflammatory bowel disease may exhibit inadequate or adverse responses to these medications, potentially attributable to the activation of alternative biological pathways in intestinal inflammation. Consequently, the exploration of novel therapeutic agents is imperative (8). It is widely acknowledged that the development of each new drug necessitates substantial financial and temporal investments (9). Investigating the efficacy of established pharmaceuticals offers a potential solution to mitigate this concern, as the mechanisms of action of these drugs are well-established.

**Figure 1:**
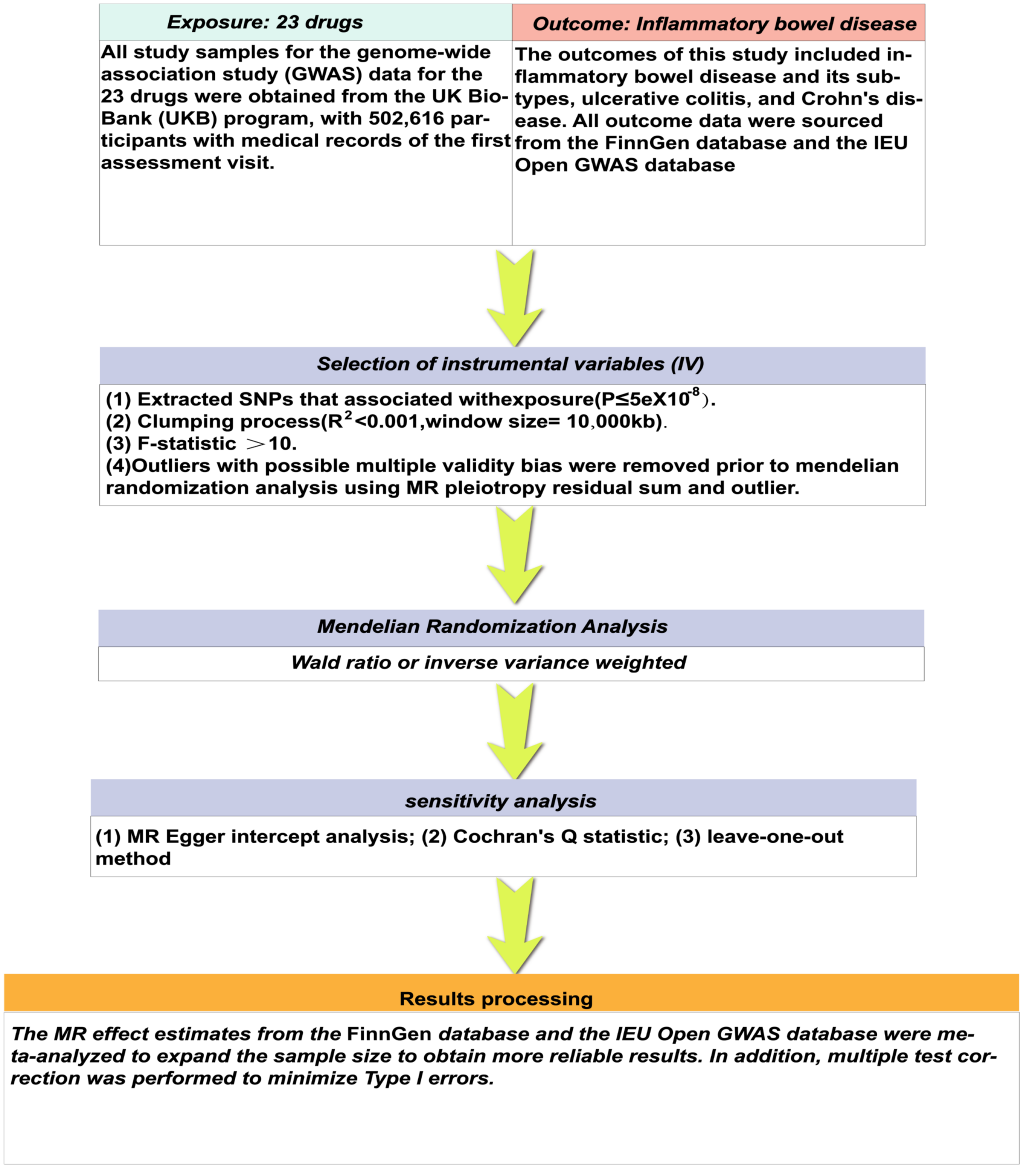
Overview of the research design. The figure was generated using Adobe illustrator.

Mendelian randomization analysis presents a valuable substitute for traditional randomized clinical trials, utilizing genetic variations to deduce causal associations between exposure and outcome (10). In comparison to randomized controlled trials (RCTs), MR analysis can reduce potential biases inherent in trial design and execution, as well as balance the effects of confounding factors (11, 12). This study employed two-sample Mendelian randomization to evaluate the causal association between various medications and IBD, utilizing data from recent drug genome-wide association studies across diverse populations to identify drugs with potential correlations. Subsequently, these drugs can be investigated in clinical trials to offer insights for future drug trials (Figure 1).

## Method

### Basic assumptions and study design of MR

MR studies rely on strict adherence to three assumptions: (1) the assumption of relevance (13), that instrumental variables are strongly associated with inflammatory bowel disease; (2) the assumption of independence, that instrumental variables affect outcomes only through their effect on inflammatory bowel disease and not through any alternative causal pathway; and (3) the assumption of exclusionary restrictions (14), that instrumental variables should not be directly associated with inflammatory bowel disease. The design of this study was informed by the Mendelian Report Card for Enhanced Observational Epidemiological Studies by Randomization (STROBE-MR) (12), and all populations in this study were drawn from subjects of European ancestry to reduce population stratification bias. In addition, all data used in this work were obtained from studies with subject consent and ethical approval; therefore, our study did not require ethical approval from an institutional review board.

### Data sources

All study samples for the GWAS data for the 23 drugs were obtained from the UK BioBank (UKB) program,^1^ with 502,616 participants with medical records of the first assessment visit (15). The 23 classes of drugs were derived from the 6,745 drugs included in the UKB and were subjected to multiple quality checks to generate the final pooled GWAS data. We obtained GWAS data for these 23 drugs, including immunosuppressants, diabetes drugs, β-blockers, etc. Gene-outcome associations for IBD were obtained from two independent databases: (1) FinnGen database^2^ (2); IEU Open GWAS database.^3^ In the FinnGen study, the number of cases and controls was 5673/213,119 for IBD, 2251/210,300 for UC, and 657/210,300 for CD, respectively. In the IEU study, the number of cases and controls of IBD was 25,042/34915, UC 12366/33609, and CD 12194/28072, respectively. The GWAS data for exposures and outcomes came from three largely independent samples, so there was essentially no sample overlap nor impact on the study results (Supplementary Documents S1, S2).

### Instrumental variable selection

To obtain reliable IVs, not only did we strictly adhere to the three assumptions mentioned earlier (13), but we also used a series of stringent criteria: (1) selecting SNPs with genome-wide significance (*p* < 5 × 10^−8^) along with an acceptable probability of mutation (minor allele frequency > 1%); (2) performing clump (r^2 < 0.001, kb = 10,000 kb) to eliminate linkage disequilibrium between IVs, (3) removing palindromic SNPs when present, (4) the F statistic was used to estimate the strength of each genetic tool and select all strong tool variables (*F* > 10). Equation (16) is R2 × (N-2)/(1-R2), where R2 is the cumulative explained variance of the selected SNPs in the exposure and N is the number of samples. Subsequently, we utilized MR pleiotropy residual sum and outlier (MR PRESSO) to exclude outliers with possible polytropic bias before MR analysis (Supplementary Document S9).

### MR analysis

In this study, we used five different methods for two-sample MR analysis, each making different assumptions about the validity of IVs. Random effects model inverse variance weighting (Re-IVW) is considered the most concise and reliable method for MR analysis, which is used to combine the causal effects of individual SNPs, allow for heterogeneity between SNPs, and return unbiased estimates of causality when all IVs are valid and the level of pleiotropy is balanced (17). Therefore, we used it as the primary analysis method. The other four sensitivity analysis methods (MR Egger, weighted median method, simple modal method, and weighted modal method) were used as complementary to the results. In addition, we performed a meta-analysis of the MR effect estimates for each of the two cohorts to expand the sample size to obtain more reliable results. In addition, to minimize Type I errors, we performed a multiple testing correction. Finally, we also used the Egger intercept test to assess the presence of horizontal pleiotropy and the Cochrane’s Q test to assess heterogeneity among the included SNPs. Among them, there is statistical significance when the P of FDR, pleiotropy, and heterogeneity are less than 0.05.

All of the above analyses were performed on R software version 4.2.2. The R package TwoSampleMR was used to perform MR analysis, the R package MR-PRESSO was used to perform MR-PRESSO, and the R package meta performed meta-analysis.

## Results

### Causal risk relationship between 23 drugs and IBD

**Figure 2:**
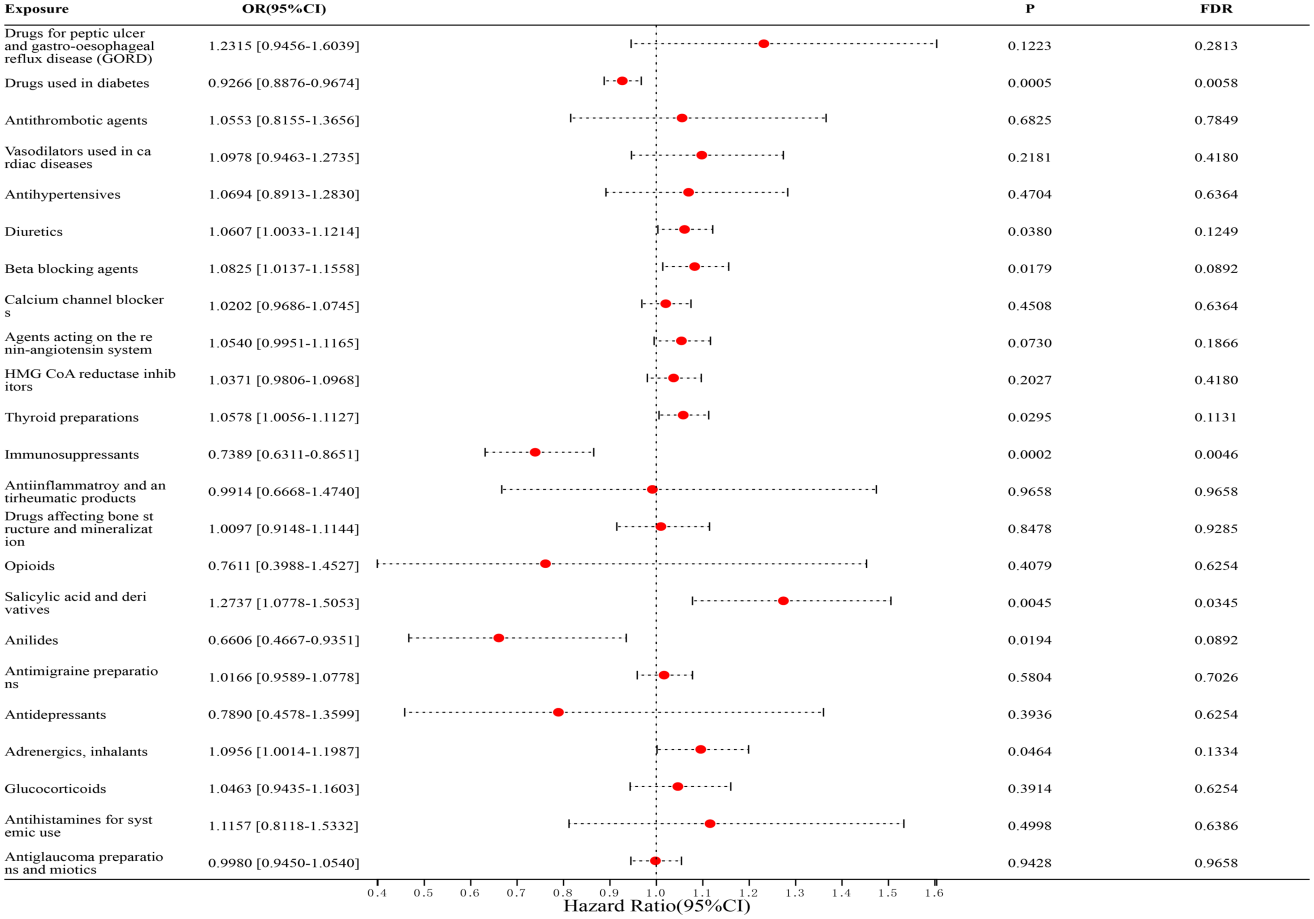
Forest map of 23 drugs associated with IBD risk. FDR is the *p*-value corrected by multiple tests.

In our study of 23 classes of drugs associated with IBD, we found a significant correlation between diabetic drugs, immunosuppressants, salicylic acid, and derivatives and the risk of IBD. Among them, diabetes medications (FinnGen dataset: OR = 0.9289, [95% CI, 0.8729–0.9886], *p* = 0.1559; IEU dataset: OR = 0.9245, [95% CI, 0.8709–0.9814], *p* = 0.0765; combined estimate: OR = 0.9266, [95% CI, 0.8876–0.9674], *p* = 0.0058) and immunosuppressants (FinnGen dataset: OR = 0.7829, [95% CI, 0.6434–0.9526], *p* = 0.1559; IEU dataset: OR = 0.6652, [95% CI, 0.5105–0.8667], *p* = 0.0582; combined estimate: OR = 0.7389, [95% CI, 0.6311–0.8651], *p* = 0.0046) were negatively associated with the risk of IBD; salicylic acid and derivatives (FinnGen dataset: OR = 1.2862, [95% CI, 0.9354–1.7685], *p* = 0.4652; IEU dataset: OR = 1.2690, [95% CI, 1.0429–1.5441], *p* = 0.0855; combined estimate: OR = 1.2737, [95% CI, 1.0778–1.5053], *p* = 0.0345) was positively associated with the risk of IBD. The detailed results are shown in Figure 2.

### Causal risk relationship between 23 drugs and UC

**Figure 3:**
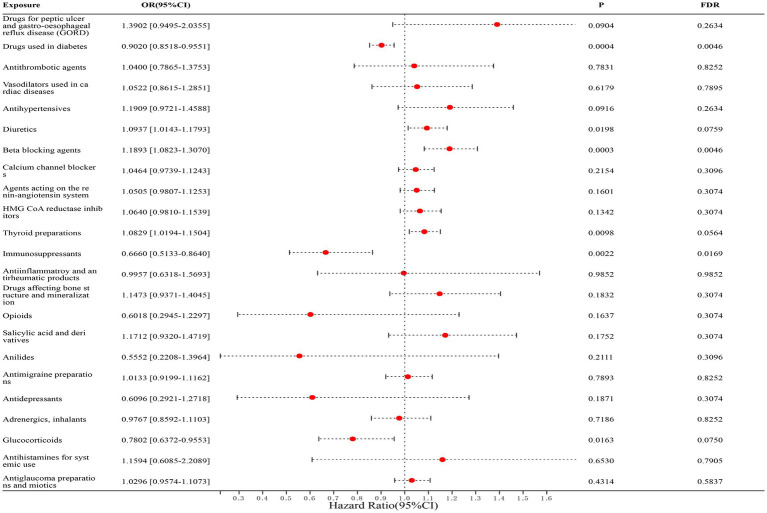
Forest map of 23 drugs associated with UC risk. FDR is the *p*-value corrected by multiple tests.

In correlation studies with UC, we found that diabetes medications (FinnGen dataset: OR = 0.8909, [95% CI, 0.8066–0.9840], *p* = 0.1309; IEU dataset: OR = 0.9075, [95% CI, 0.8461–0.9733], *p* = 0.0758; combined estimate: OR = 0.9020, [95% CI, 0.8518–0.9551], *p* = 0.0046) and immunosuppressants (FinnGen dataset: OR = 0.6696, [95% CI, 0.5064–0.8853], *p* = 0.0910; IEU dataset: OR = 0.6426, [95% CI, 0.3130–1.3195], *p* = 0.4376; combined estimate: OR = 0.6660, [95% CI, 0.5133–0.8640], *p* = 0.0169) were significantly associated with a reduced risk of UC; whereas β-blockers (FinnGen dataset: OR = 1.1719, [95% CI, 0.9818–1.3989], *p* = 0.2596; IEU dataset: OR = 1.1963, [95% CI, 1.0701–1.3373], *p* = 0.0373; Combined estimate: OR = 1.1893, [95% CI, 1.0823–1.3070], *p* = 0.0046) was significantly associated with an increased risk of UC. The detailed results are shown in Figure 3.

### Causal risk relationship between 23 drugs and CD

**Figure 4:**
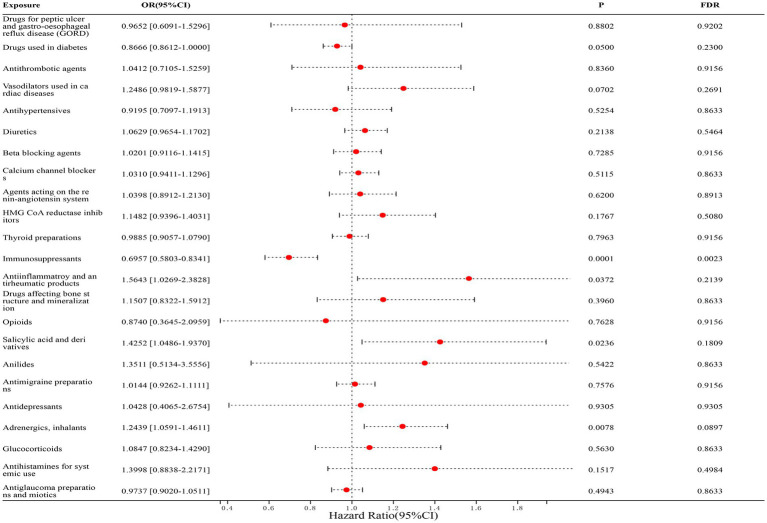
Forest map of 23 drugs associated with CD risk. FDR is the *p*-value corrected by multiple tests.

In the correlation studies with CD, we found that only immunosuppressants (FinnGen dataset: OR = 0.6866, [95% CI, 0.5426–0.8688], *p* = 0.0401; IEU dataset: OR = 0.7093, [95% CI, 0.5336–0.9429], *p* = 0.2253; combined estimates: OR = 0.6957, [95% CI, 0.5803–0.8341], *p* = 0.0023) was significantly associated with a reduced risk of CD, and the remaining drugs were not statistically associated with CD. The detailed results are shown in Figure 4.

### Sensitivity analysis

The results of our MR analysis indicated that four drugs, diabetes medications, immunosuppressants, salicylic acid and derivatives, and β-blockers, were associated with IBD and its subtypes (UC and CD). Although there was heterogeneity in Cochran’s heterogeneity Q test for immunosuppressants among the four drugs, the heterogeneity was acceptable given that we used random effects IVW as the primary outcome. Moreover, we had already eliminated SNPs with possible pleiotropy by the MR-PRRESSO method before MR analysis, and we did not show any signs of pleiotropic effects in the MR-Egger method for assessing the presence of horizontal pleiotropy (Supplementary Documents S3–S8). Therefore, the results of our study are reliable.

## Discussion

This is the first MR study to investigate whether multiple drugs are associated with the risk of IBD and its subtypes. In recent years, there has been a notable increase in the incidence rates of not only IBD but also other chronic conditions such as diabetes and cardiovascular disease, leading to a growing number of individuals experiencing multiple comorbidities (3, 18, 19). The treatment methods are all personalized based on specific diseases unless there are contraindications between drugs. So, the potential impact of drugs on comorbid conditions is often overlooked, leading to missed opportunities for utilizing existing drugs in novel ways. Our results to some extent fill this gap. In our study, we found that a total of 4 drugs were associated with increased or decreased risk of IBD and its subtypes (UC and CD), while no significant correlation was found between the other 19 drugs and inflammatory bowel disease or its subtypes. Among them, immunosuppressants are a common protective factor for IBD and its subtypes (UC and CD); Diabetes drugs are protective factors for IBD and UC; β-blockers are a risk factor for UC; Salicylic acid and its derivatives are risk factors for IBD.

Ulcerative colitis (20), a significant subtype of inflammatory bowel disease, is characterized by chronic, nonspecific inflammation primarily affecting the colorectal mucosa, and has been identified as a modern refractory disease by the World Health Organization. The conventional treatment options for patients diagnosed with ulcerative colitis typically include corticosteroids (21), aminosalicylates (22), and immunosuppressive agents (23). Immunosuppressants, in particular, are commonly prescribed for long-term remission maintenance in individuals with mild to moderate disease who do not respond well to 5-ASA, those who are dependent on steroids, and those who have shown positive responses to cyclosporine or tacrolimus (24). T-cells (25) are the key molecules responsible for mucosal damage in UC, especially in the CD4Th2 phenotype (26). Immunosuppressants have shown efficacy in treating ulcerative colitis by inhibiting the proliferation and function of cytotoxic T-cells and natural killer cells, inducing apoptosis, and exerting direct anti-inflammatory effects (27). Furthermore, numerous observational studies have shown that commonly utilized hypoglycemic agents such as metformin and sulfonylureas exhibit therapeutic promise in the management of UC (28, 29). Metformin, classified as a biguanide drug, can modulate various pathways including TGF-β (30), NF-κB (31), LKB1/AMPK, and JNK (32, 33), thereby safeguarding the integrity of the intestinal barrier, preserving normal mitochondrial structure, and mitigating inflammation in intestinal tissues. These mechanisms contribute to the potential therapeutic efficacy of metformin in the treatment of UC. Gliclazide (29), a member of the sulfonylurea class of drugs, has demonstrated efficacy in mitigating intestinal inflammation in rats with ulcerative colitis through modulation of PPARγ, NF-κB, and MAPK signaling pathways in preclinical studies. These findings align with the protective factors identified in our Mendelian analysis. β-Blockers function as antagonists of β-adrenergic receptors (ARs), which are crucial in regulating physiological functions including blood pressure, heart rate, and respiratory tract function (34). Despite the lack of direct correlation studies between β-blockers and ulcerative colitis, there is significant evidence suggesting that PKA phosphorylation of β-2AR can lead to the coupling of the receptor to GAI, resulting in the inhibition of cAMP production via AC. This inhibition triggers the GBG/PI3K/protein kinase B (Akt) cascade signaling pathway, ultimately promoting the increased expression and secretion of inflammatory factors that contribute to colonic mucosal injury (35, 36). Our Mendelian results indicate a potential correlation and offer valuable insights for future research and development in this area.

Crohn’s disease, a significant subtype of inflammatory bowel disease, is a chronic inflammatory granulomatous condition affecting the gastrointestinal tract with unknown etiology. It can manifest in any part of the gastrointestinal tract, presenting with symptoms such as abdominal pain, weight loss, and altered bowel patterns (37). Due to the ongoing investigation into the pathogenesis of CD, there is currently no definitive cure (38). The drugs currently used to treat Crohn’s disease are mainly nonbiological agents (anti-inflammatory drugs, steroids, immunosuppressants) and biological therapies (anti-tumor necrosis factor, anti-tumor α4β7 Integrins, Antibiotics α- Integrins and anti interleukins12/23) (39). Whereas the results of our study related to CD were immunosuppressive agents, which corroborates the traditional drugs. A reduction in the apoptotic rate of T cells within the intestinal lamina propria may contribute to inflammation in individuals with Crohn’s disease. In patients with Crohn’s disease who respond to azathioprine, both azathioprine and 6-mercaptopurine, which are representative immunosuppressive agents, have been demonstrated to enhance the apoptosis rate of peripheral T cells *in vitro* (40, 41). Furthermore, in addition to promoting apoptosis, the inhibition of pro-inflammatory T-cell proliferation is another mechanism through which azathioprine exerts its effects in patients with CD (42). Although our results support the protective effect of immunosuppressive agents, biologic agents are not always superior to nonbiologic agents. Each type of agent offers distinct advantages in various therapeutic approaches for Crohn’s disease, indicating a significant potential for further exploration.

Additionally, our study did not identify a correlation between inflammatory bowel disease subtypes (UC and CD) and salicylic acid and its derivatives. However, salicylic acid and its derivatives were found to be positively associated with the overall risk of inflammatory bowel disease, presenting a clinical paradox (43). The majority of clinical studies on salicylic acid and its derivatives have primarily examined their effectiveness in treating patients with inflammatory bowel disease (IBD). While these studies have shown that salicylic acid and its derivatives can alleviate clinical symptoms and improve prognosis in IBD patients, it is important to note that their use may still pose a risk for developing IBD in high-risk populations. Future research should prioritize investigating the long-term use of salicylic acid and its derivatives in high-risk groups to better understand their potential implications.

This article utilizes the most recent GWAS drug database and, for the first time, employs a large-scale GWAS database to validate the causal association between 23 drugs and IBD. The utilization of MR analysis helps mitigate confounding variables, thereby enhancing the credibility of the findings compared to observational studies (13). Our study offers novel strategies for the prevention and management of IBD, while also suggesting avenues for future clinical investigations. Nonetheless, it is important to acknowledge the limitations of our research. The exposed GWAS data provides 23 types of drugs and cannot be precise to a specific drug. For example, in our results, hypoglycemic drugs are a protective factor for inflammatory bowel disease, while hypoglycemic drugs include biguanides, sulfonylureas, thiazolidinediones α- Various drugs such as glucosidase inhibitors, and GLP-1 receptor agonists (44). Different hypoglycemic drugs have different mechanisms, which means that the effects of different hypoglycemic drugs on inflammatory bowel disease are different. Consequently, it is not appropriate to make broad generalizations regarding the efficacy of hypoglycemic drugs in treating inflammatory bowel disease. Moving forward, there is a necessity for a more precise and comprehensive categorization of drug genome-wide association study data. Additionally, dual sample Mendelian randomization analysis serves as a tool solely capable of offering estimations of potential causal relationships, rather than confirming direct causal effects of the 23 drugs on inflammatory bowel disease. Therefore, further investigation is warranted.

## Data availability statement

The original contributions presented in the study are included in the article/Supplementary material, further inquiries can be directed to the corresponding authors.

## Ethics statement

Ethical approval was not required for the study involving humans in accordance with the local legislation and institutional requirements. Written informed consent to participate in this study was not required from the participants or the participants’ legal guardians/next of kin in accordance with the national legislation and the institutional requirements.

## Author contributions

LH: Software, Writing – original draft. TD: Investigation, Methodology, Software, Writing – review & editing. YH: Data curation, Writing – review & editing. WY: Software, Writing – review & editing. JY: Conceptualization, Funding acquisition, Supervision, Writing – review & editing. GS: Conceptualization, Supervision, Writing – review & editing.

## Footnotes

^1^ https://www.ukbiobank.ac.uk/

^2^ https://www.finngen.fi/en

^3^ https://gwas.mrcieu.ac.uk/datasets
