# Supplementary material for: Association between 23 drugs and inflammatory bowel disease: a two-sample Mendelian randomization study
Source: Front Med (Lausanne). 2024 May 21;11:1371362. doi: 10.3389/fmed.2024.1371362 (PMC11149542; doi:10.3389/fmed.2024.1371362)
Supplement: Supplementary file 3 [file Data_Sheet_1.doc]

**Leave-one-out analysis**

**The splicing diagram of leave-one-out analysis in FinnGen database.**
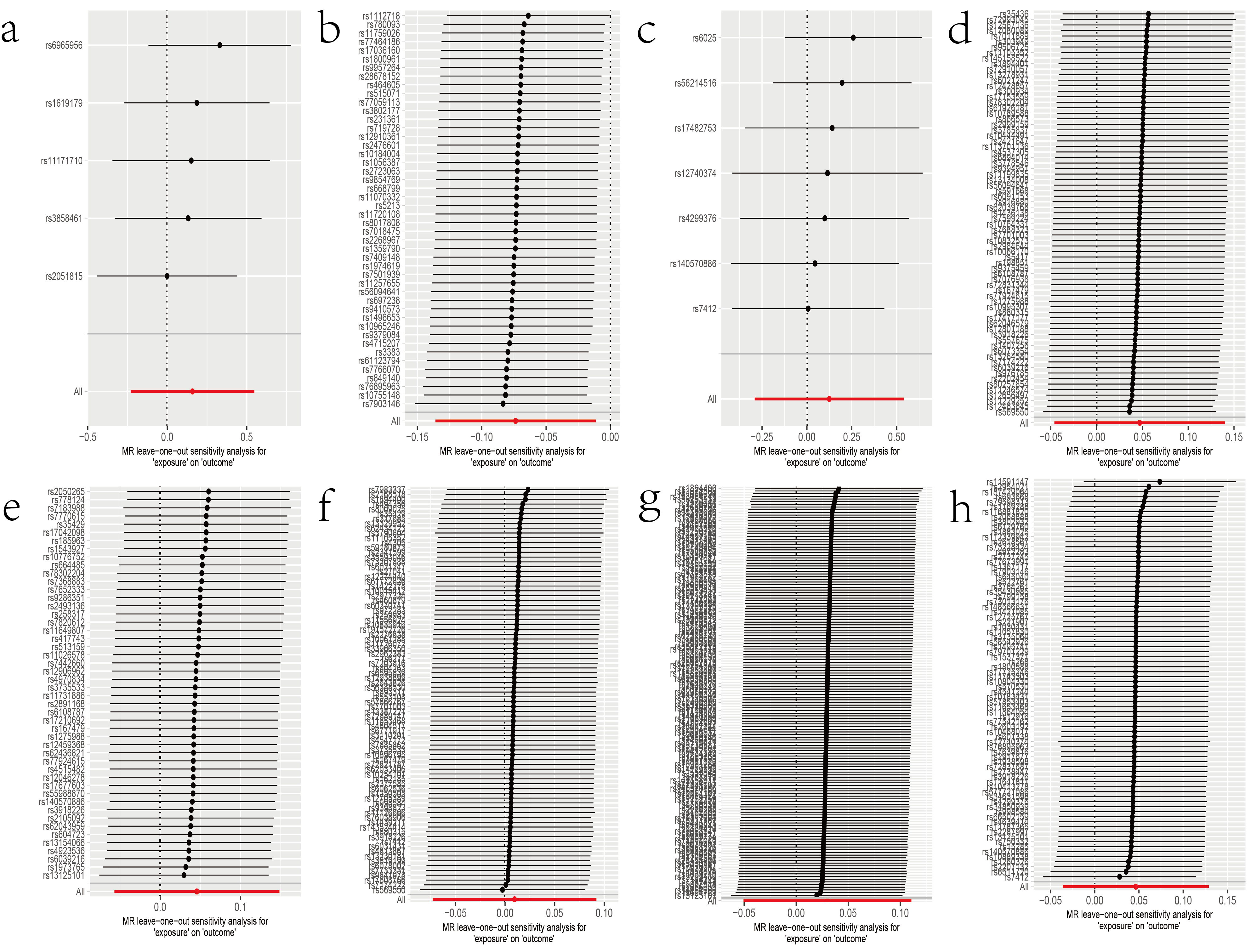


Figure 1Note: A splicing diagram of 8 drugs and IBD from the FinnGen database.**a** represents Drugs “for peptic ulcer and gastro-oesophageal reflux disease”; **b** represents “Drugs used in diabetes; **c** represents Antithrombotic agents”; **d** represents “Diuretics; **e** represents Beta blocking agents”; **f** represents “Calcium channel blockers”; **g** represents “Agents acting on the renin-angiotensin system”; **h** represents “HMG CoA reductase inhibitors”.


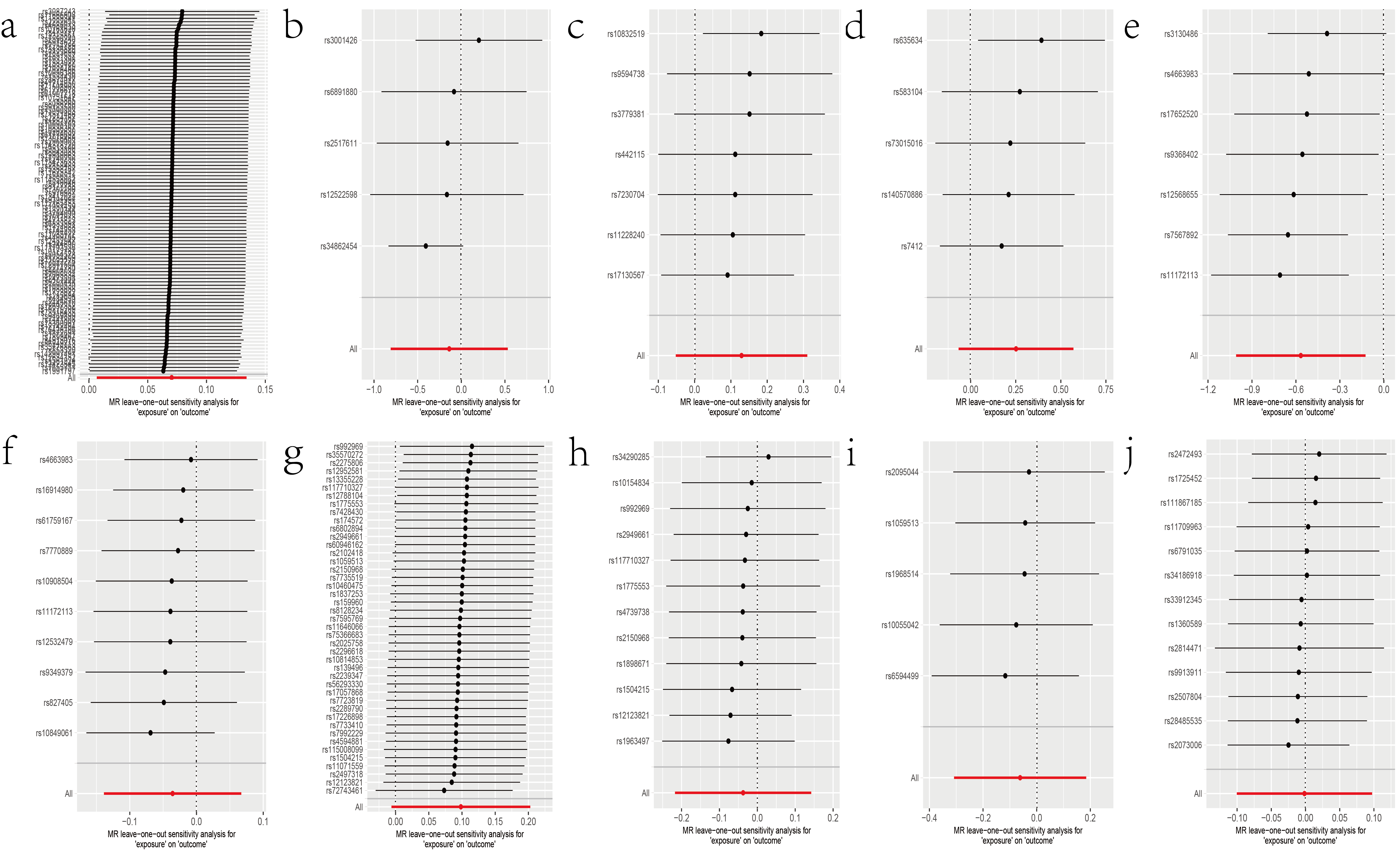


Figure 2

Note: A splicing diagram of 10 drugs and IBD from the FinnGen database.**a** represents “Thyroid preparations”; **b** represents “Anti-inflammatory and antirheumatic products, non-steroids”; **c** represents “Drugs affecting bone structure and mineralization”; **d** represents “Salicylic acid and derivatives”; **e** represents “Anilides”; **f** represents “Antimigraine preparations”; **g** represents “Adrenergics,inhalants”; **h** represents “Glucocorticoids”; **i** represents “Antihistamines for systemic use”; **j** represents “Antiglaucoma preparations and miotics”.


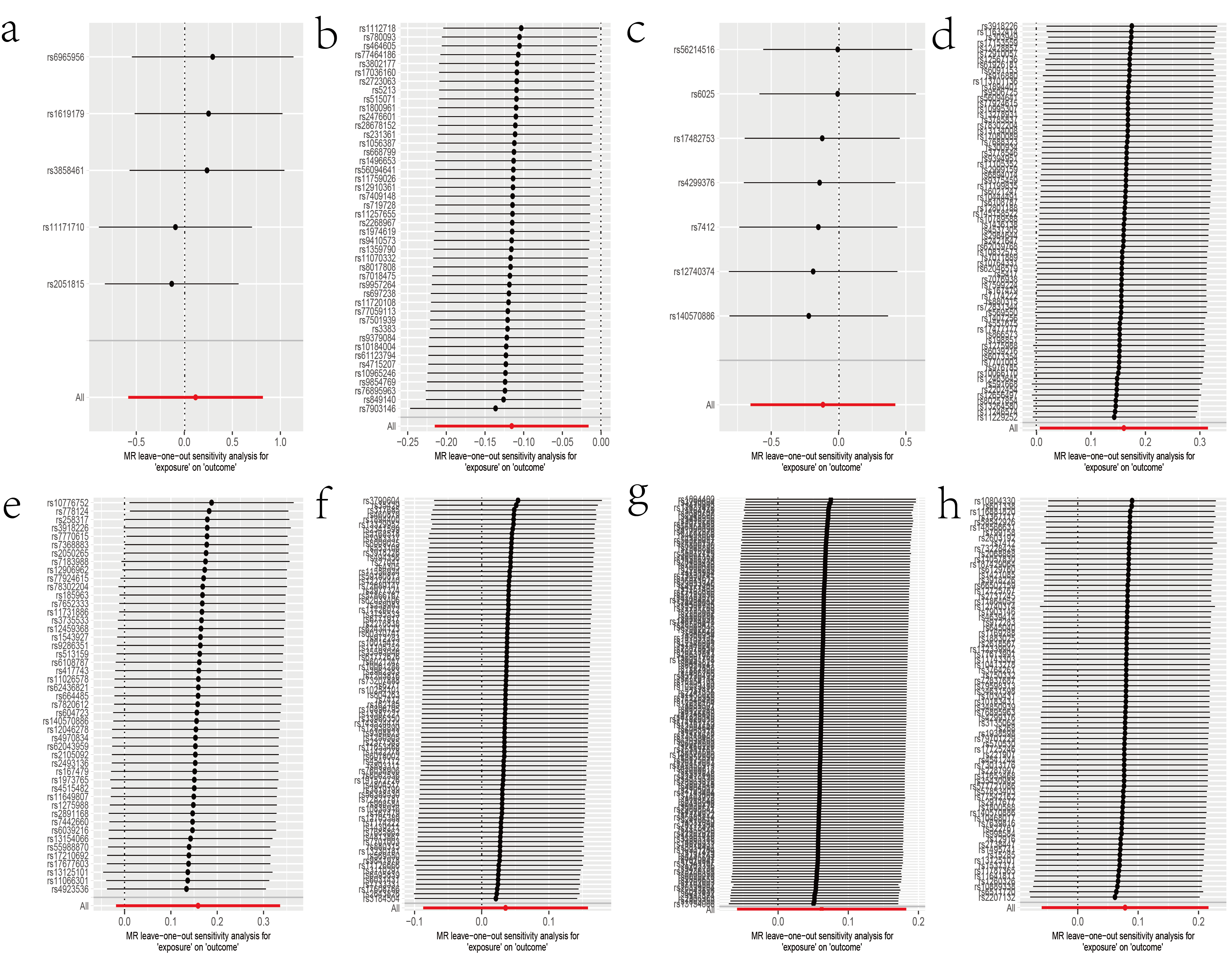


Figure 3

Note: A splicing diagram of 8 drugs and UC from the FinnGen database.**a** represents Drugs “for peptic ulcer and gastro-oesophageal reflux disease”; **b** represents “Drugs used in diabetes; **c** represents Antithrombotic agents”; **d** represents “Diuretics; **e** represents Beta blocking agents”; **f** represents “Calcium channel blockers”; **g** represents “Agents acting on the renin-angiotensin system”; **h** represents “HMG CoA reductase inhibitors”.


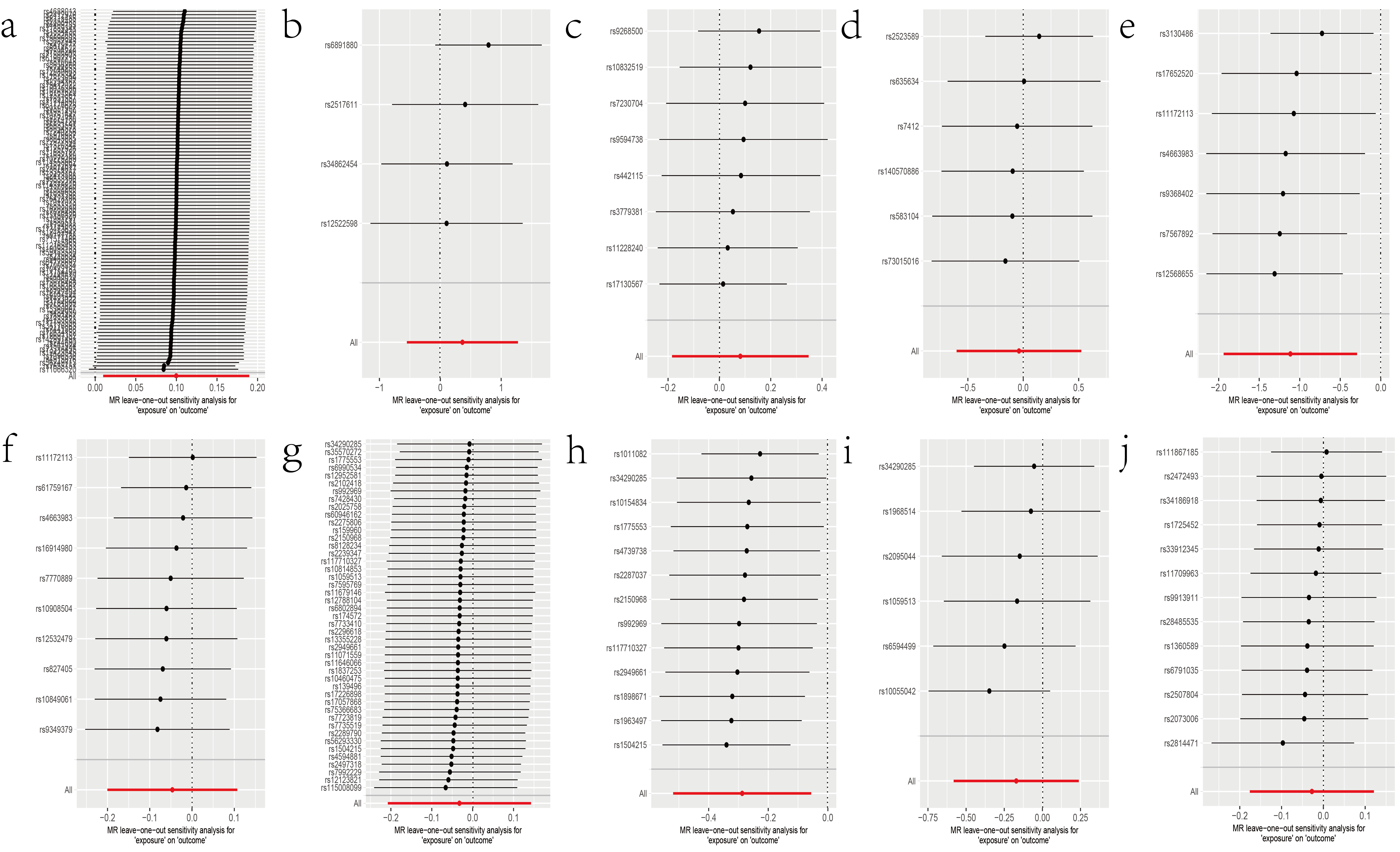


Figure 4

Note: A splicing diagram of 10 drugs and UC from the FinnGen database.**a** represents “Thyroid preparations”; **b** represents “Anti-inflammatory and antirheumatic products, non-steroids”; **c** represents “Drugs affecting bone structure and mineralization”; **d** represents “Salicylic acid and derivatives”; **e** represents “Anilides”; **f** represents “Antimigraine preparations”; **g** represents “Adrenergics,inhalants”; **h** represents “Glucocorticoids”; **i** represents “Antihistamines for systemic use”; **j** represents “Antiglaucoma preparations and miotics”.


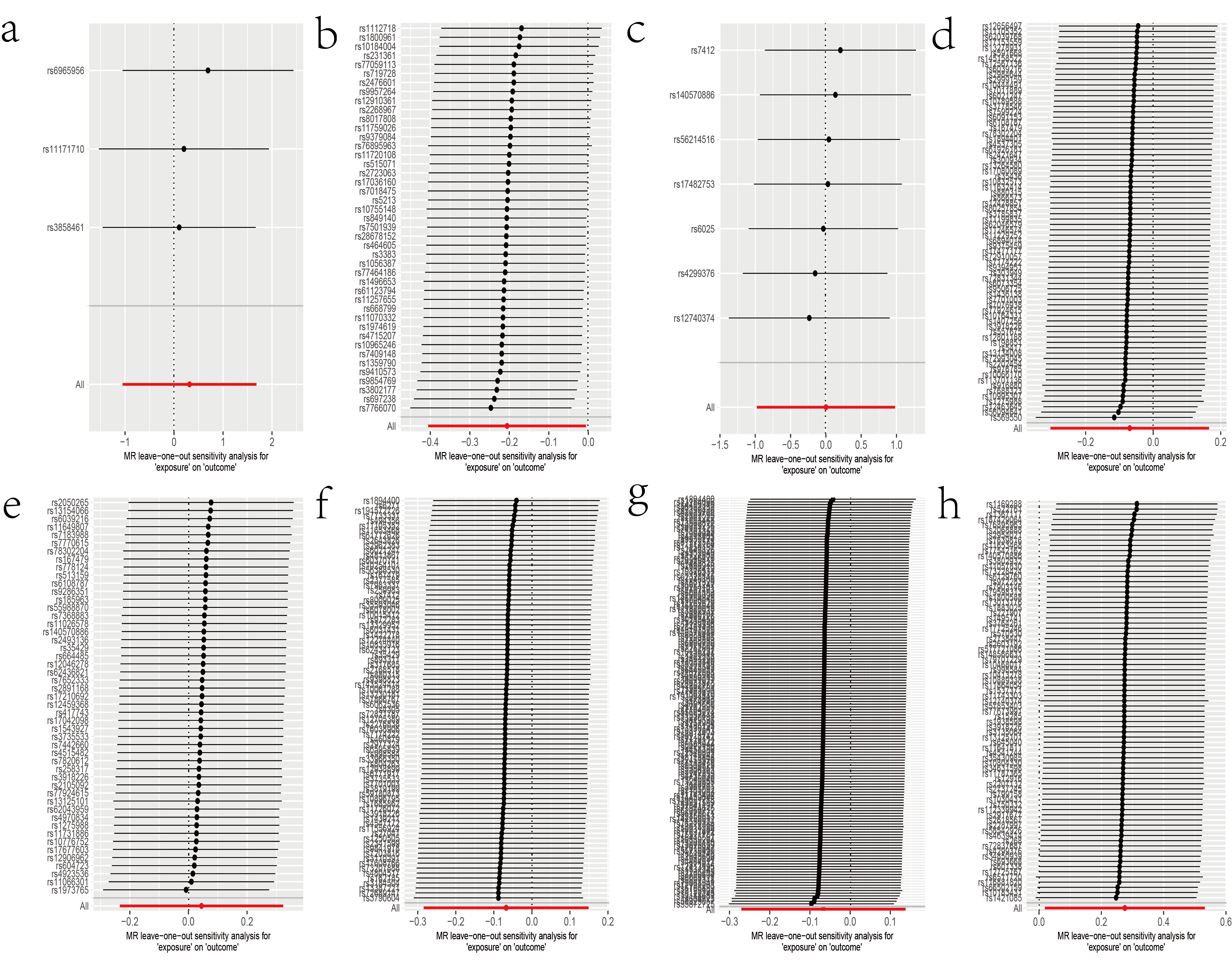


Figure 5

Note: A splicing diagram of 8 drugs and CD from the FinnGen database.**a** represents Drugs “for peptic ulcer and gastro-oesophageal reflux disease”; **b** represents “Drugs used in diabetes; **c** represents Antithrombotic agents”; **d** represents “Diuretics; **e** represents Beta blocking agents”; **f** represents “Calcium channel blockers”; **g** represents “Agents acting on the renin-angiotensin system”; **h** represents “HMG CoA reductase inhibitors”.


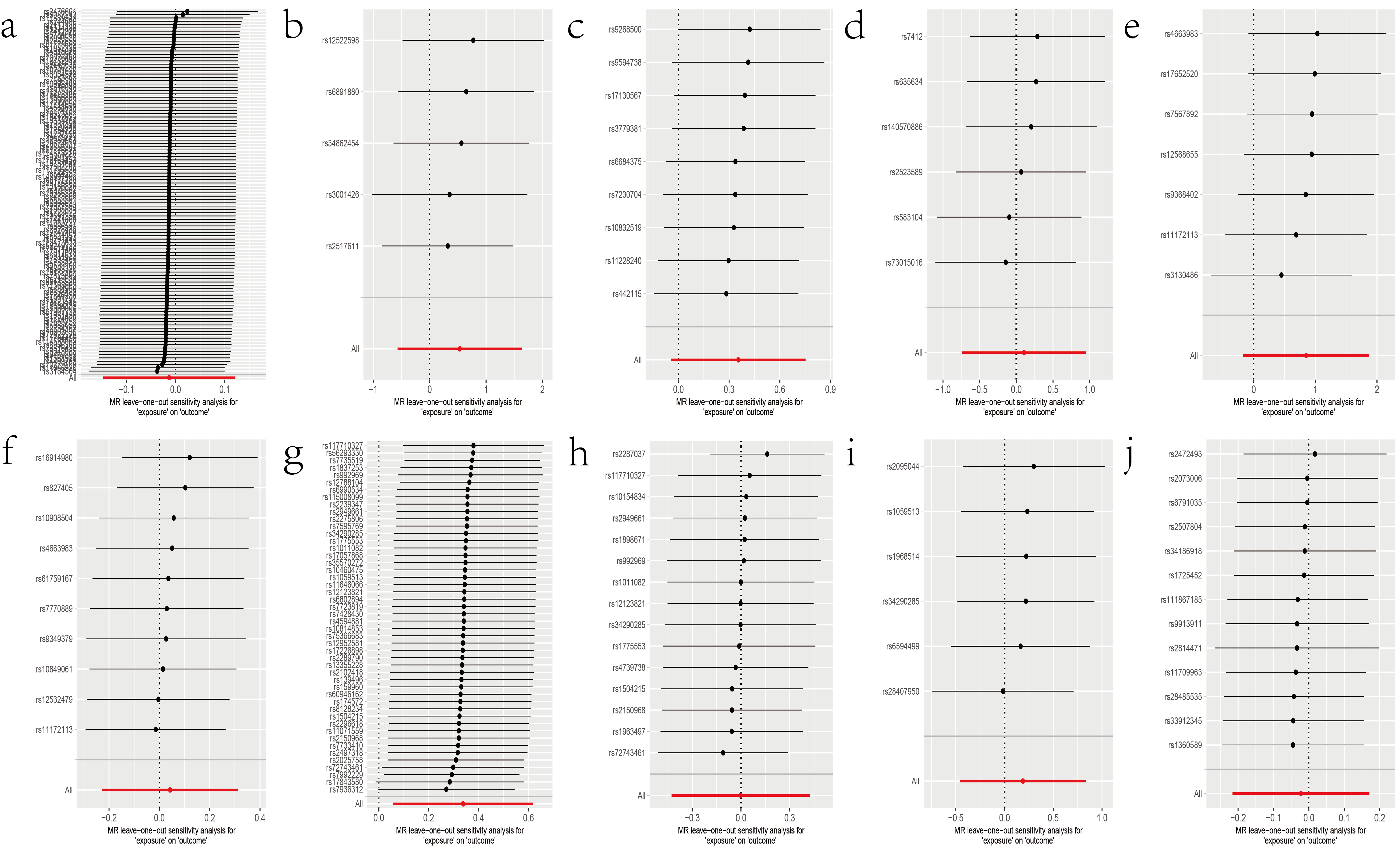


Figure 6

Note: A splicing diagram of 10 drugs and CD from the FinnGen database.**a** represents “Thyroid preparations”; **b** represents “Anti-inflammatory and antirheumatic products, non-steroids”; **c** represents “Drugs affecting bone structure and mineralization”; **d** represents “Salicylic acid and derivatives”; **e** represents “Anilides”; **f** represents “Antimigraine preparations”; **g** represents “Adrenergics,inhalants”; **h** represents “Glucocorticoids”; **i** represents “Antihistamines for systemic use”; **j** represents “Antiglaucoma preparations and miotics”.

**The splicing diagram of leave-one-out analysis in IEU database.**


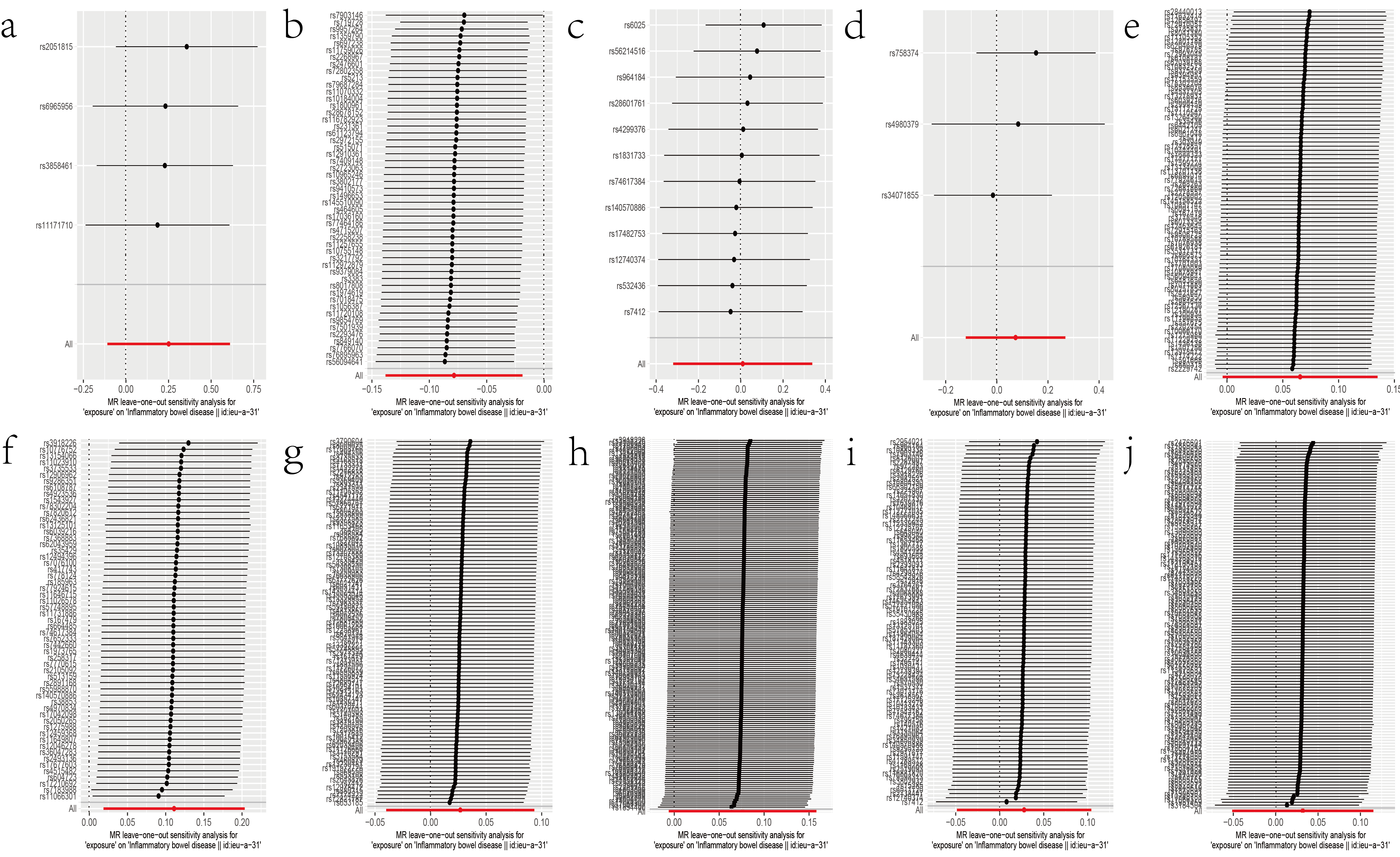


Figure 7

Note: A splicing diagram of 10 drugs and IBD from the IEU database. **a** represents “Drugs for peptic ulcer and gastro-oesophageal reflux disease”; **b** represents “Drugs used in diabetes”; **c** represents “Antithrombotic agents”; **d** represents “Antihypertensives;**e** represents Diuretics”; **f** represents “Beta blocking agents”; **g** represents “Calcium channel blockers”; **h** represents ‘Agents acting on the renin-angiotensin system”; **i** represents “HMG CoA reductase inhibitors”; **j** represents “Thyroid preparations”.


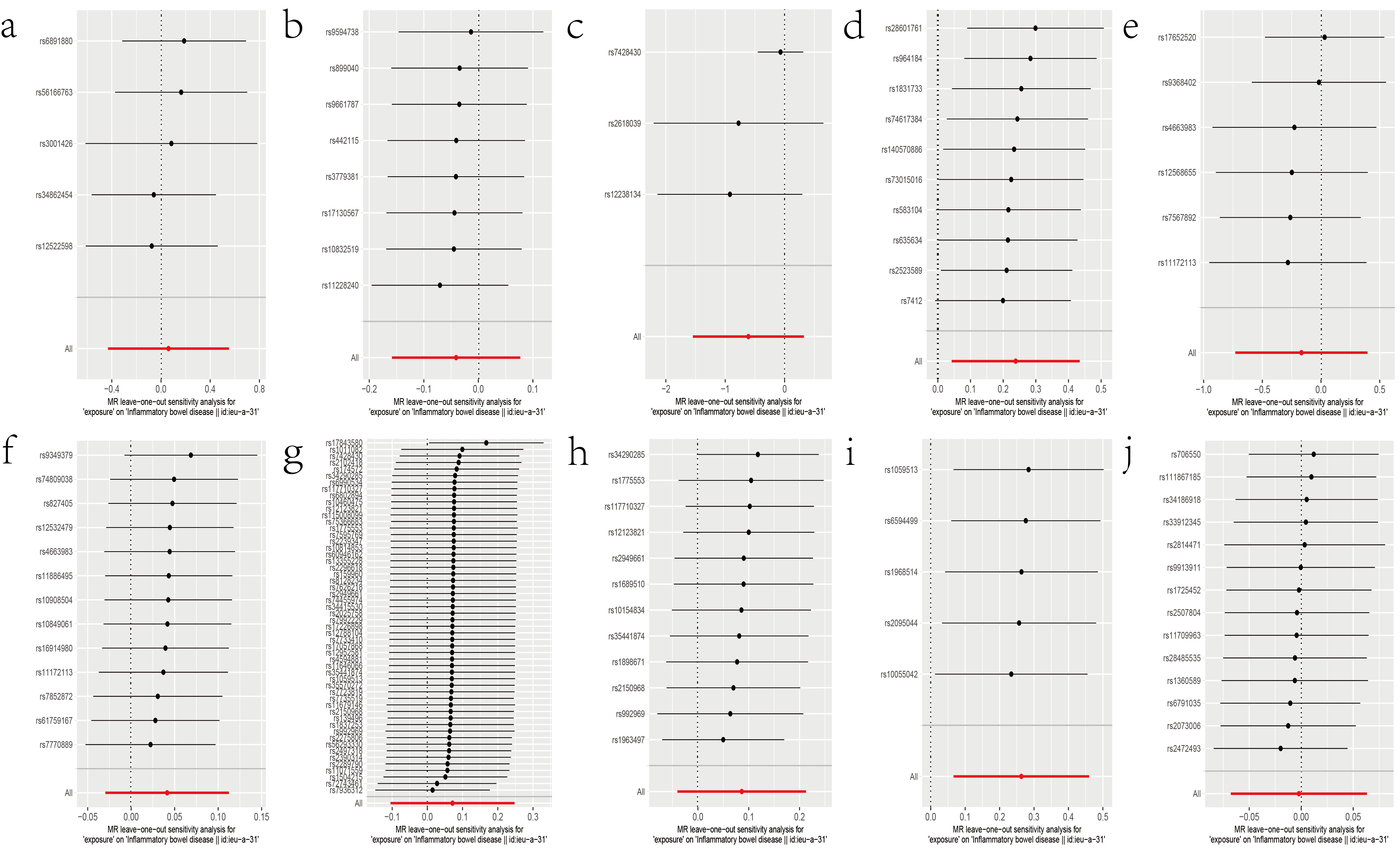


Figure 8

Note: A splicing diagram of 10 drugs and IBD from the IEU database. **a** represents “Anti-inflammatory and antirheumatic products, non-steroids”; **b** represents “Drugs affecting bone structure and mineralization”; **c** represents “Opioids”; **d** represents “Salicylic acid and derivatives”; **e** represents “Anilides”; **f** represents “Antimigraine preparations”; **g** represents “Adrenergics,inhalants”; **h** represents “Glucocorticoids”; **i** represents “Antihistamines for systemic use”; **j** represents “Antiglaucoma preparations and miotics”.


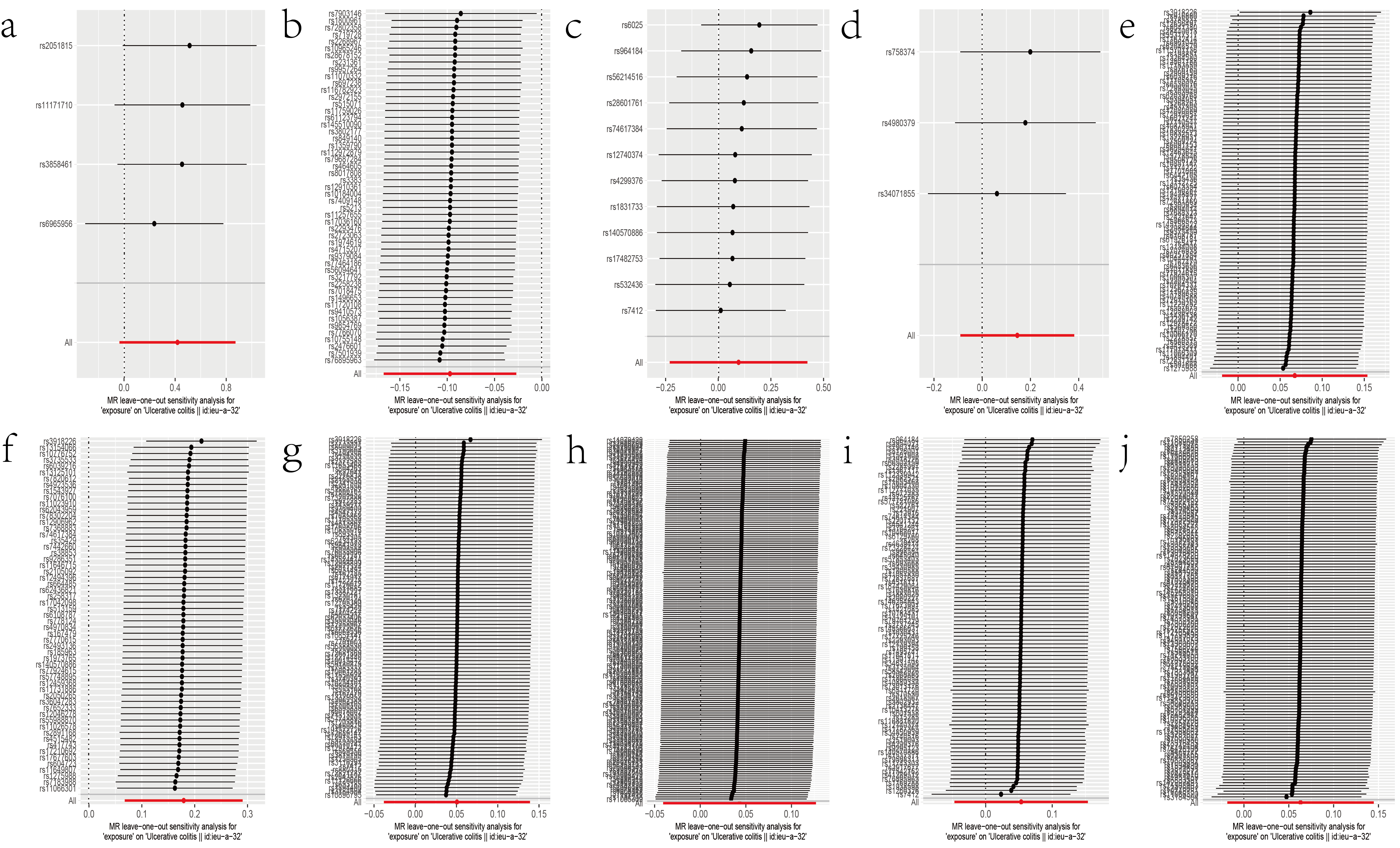


Figure 9

Note: A splicing diagram of 10 drugs and UC from the IEU database. **a** represents “Drugs for peptic ulcer and gastro-oesophageal reflux disease”; **b** represents “Drugs used in diabetes”; **c** represents “Antithrombotic agents”; **d** represents “Antihypertensives;**e** represents Diuretics”; **f** represents “Beta blocking agents”; **g** represents “Calcium channel blockers”; **h** represents ‘Agents acting on the renin-angiotensin system”; **i** represents “HMG CoA reductase inhibitors”; **j** represents “Thyroid preparations”.


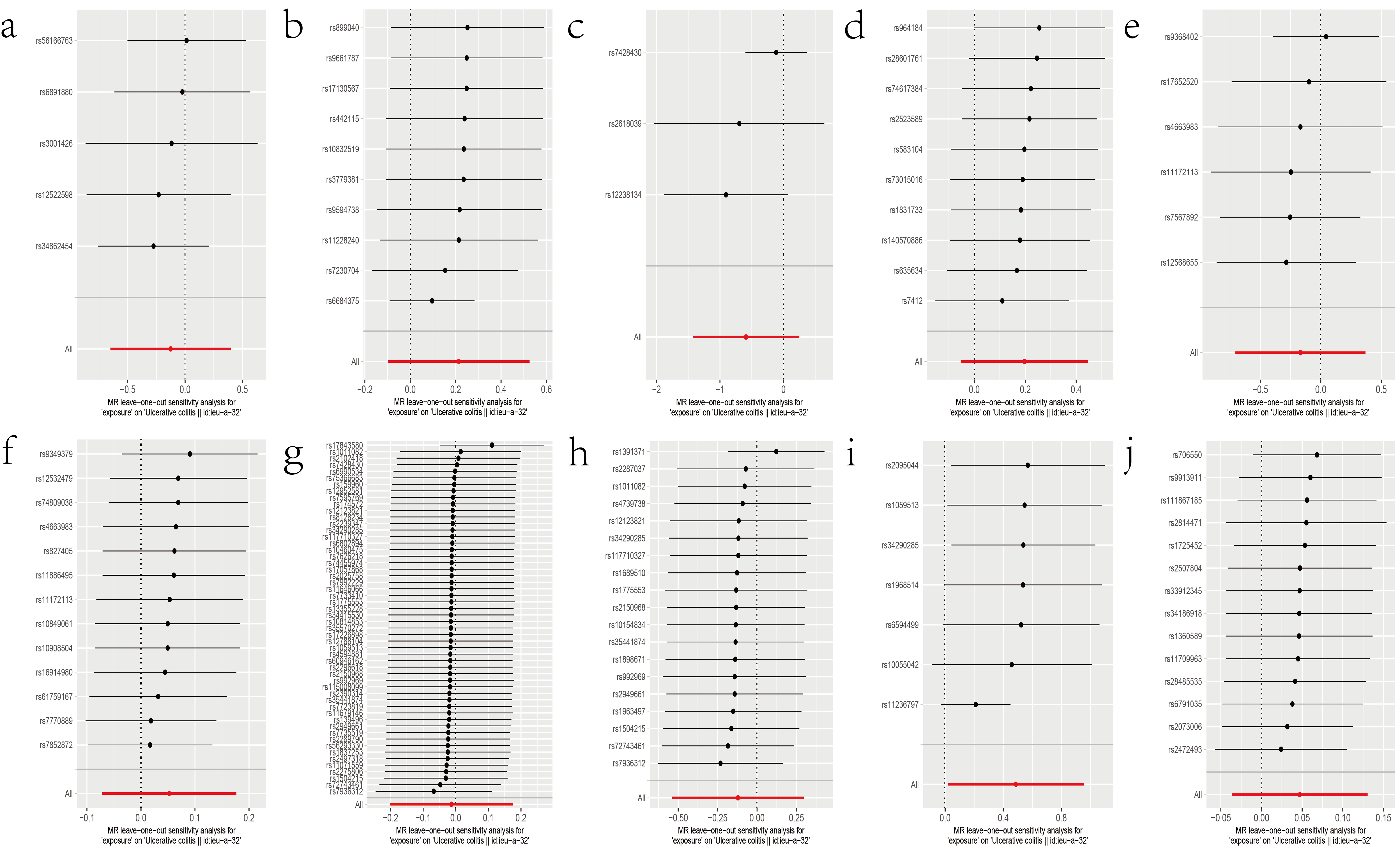


Figure 10

Note: A splicing diagram of 10 drugs and UC from the IEU database. **a** represents “Anti-inflammatory and antirheumatic products, non-steroids”; **b** represents “Drugs affecting bone structure and mineralization”; **c** represents “Opioids”; **d** represents “Salicylic acid and derivatives”; **e** represents “Anilides”; **f** represents “Antimigraine preparations”; **g** represents “Adrenergics,inhalants”; **h** represents “Glucocorticoids”; **i** represents “Antihistamines for systemic use”; **j** represents “Antiglaucoma preparations and miotics”.


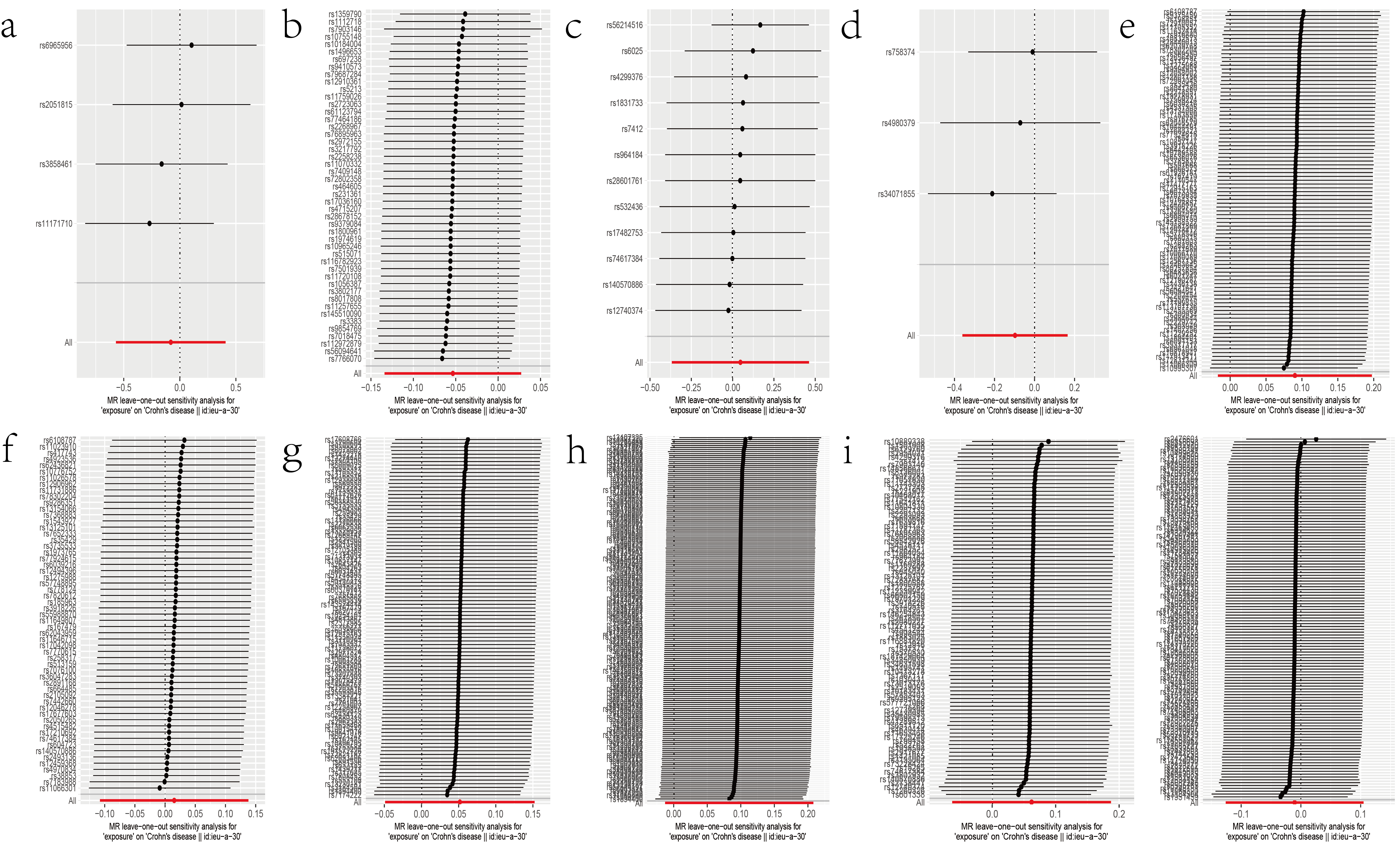


Figure 11

Note: A splicing diagram of 10 drugs and CD from the IEU database. **a** represents “Drugs for peptic ulcer and gastro-oesophageal reflux disease”; **b** represents “Drugs used in diabetes”; **c** represents “Antithrombotic agents”; **d** represents “Antihypertensives;**e** represents Diuretics”; **f** represents “Beta blocking agents”; **g** represents “Calcium channel blockers”; **h** represents ‘Agents acting on the renin-angiotensin system”; **i** represents “HMG CoA reductase inhibitors”; **j** represents “Thyroid preparations”.


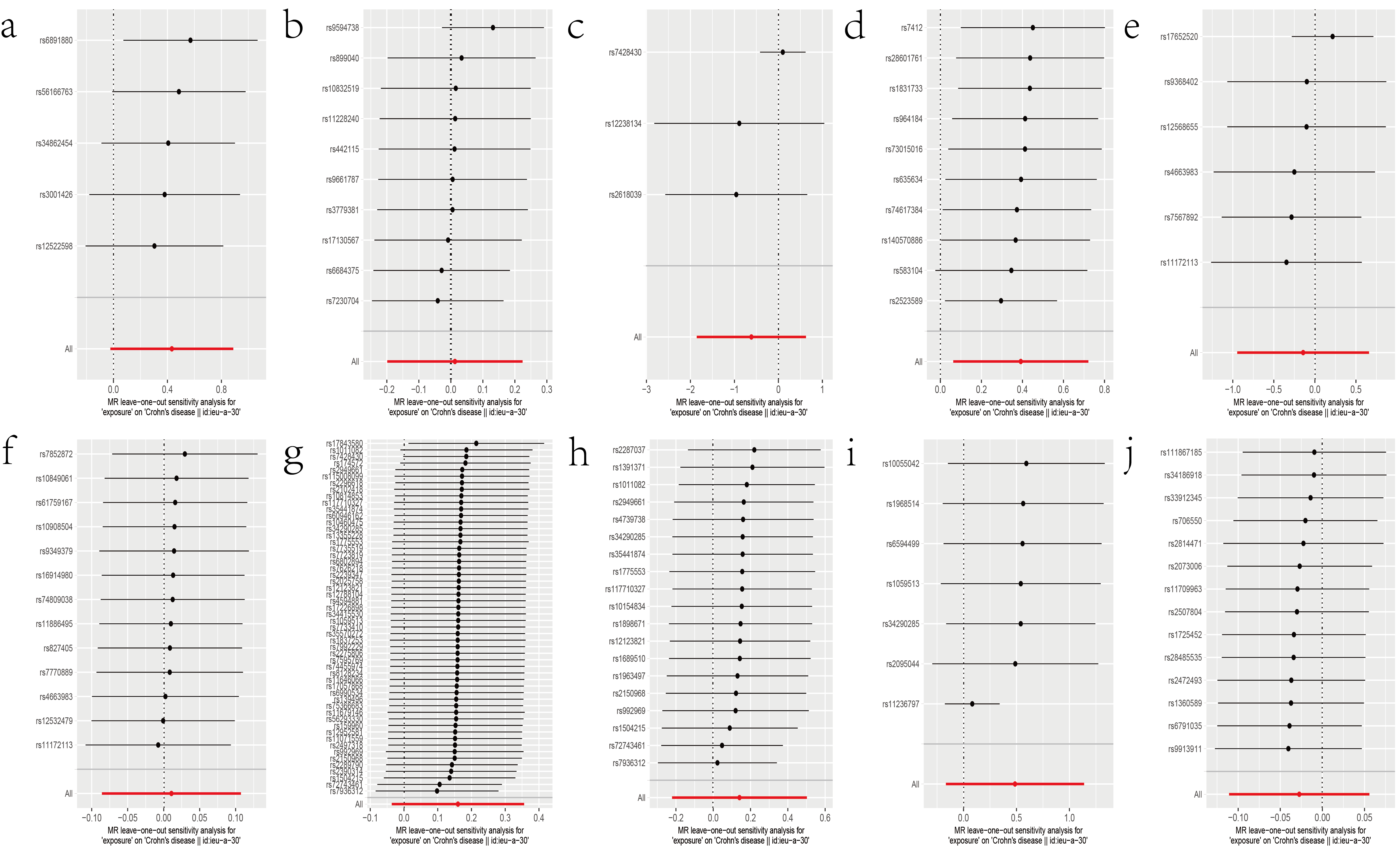


Figure 12

Note: A splicing diagram of 10 drugs and CD from the IEU database. **a** represents “Anti-inflammatory and antirheumatic products, non-steroids”; **b** represents “Drugs affecting bone structure and mineralization”; **c** represents “Opioids”; **d** represents “Salicylic acid and derivatives”; **e** represents “Anilides”; **f** represents “Antimigraine preparations”; **g** represents “Adrenergics,inhalants”; **h** represents “Glucocorticoids”; **i** represents “Antihistamines for systemic use”; **j** represents “Antiglaucoma preparations and miotics”.

Scatter plot

**The splicing diagram of scatter plots from FinnGen databases**
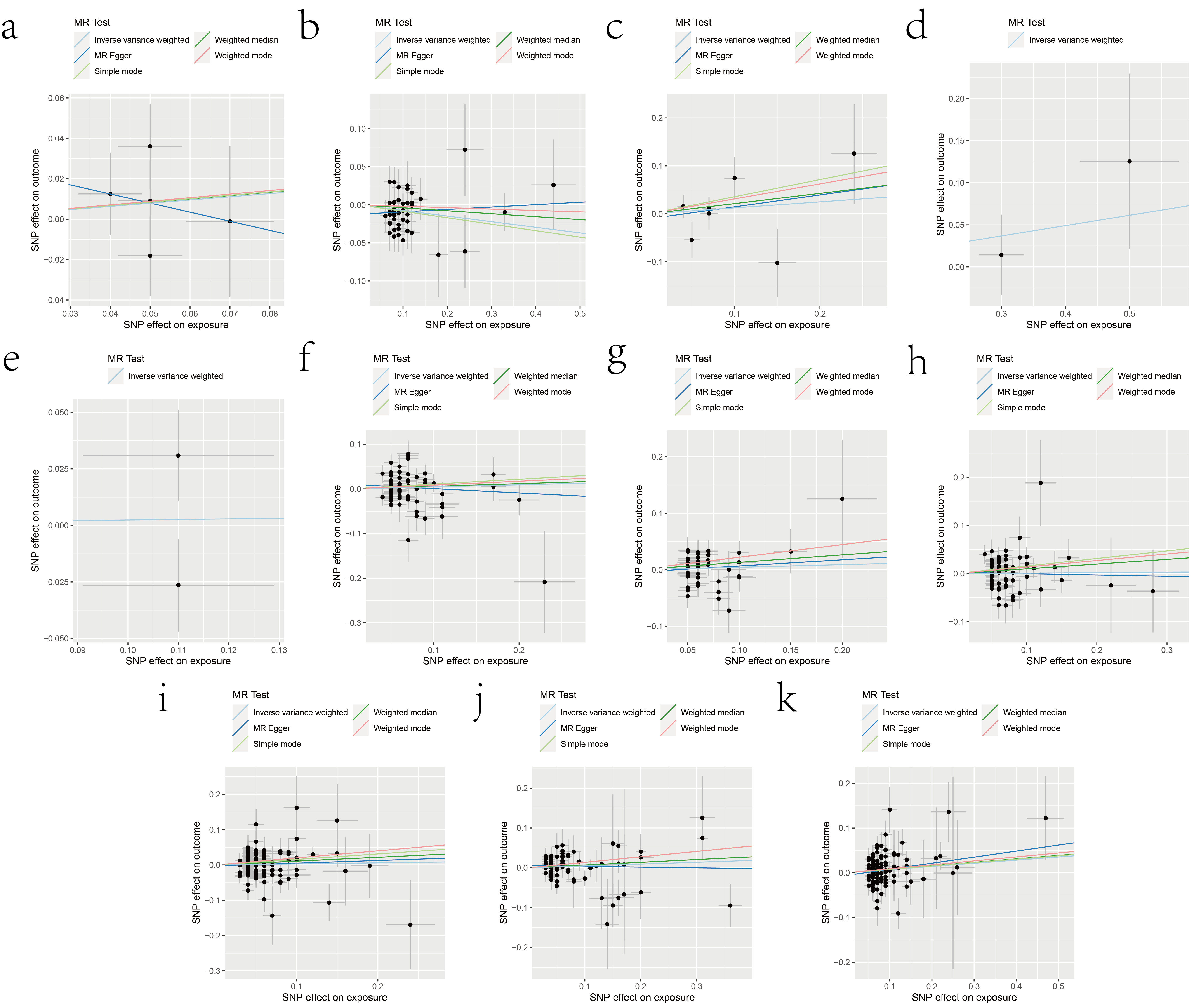


Figure 13

Note: A splicing diagram of 11 drugs and IBD from the FinnGen database.**a** represents “Drugs for peptic ulcer and gastro-oesophageal reflux disease”; **b** represents “Drugs used in diabetes”; **c** represents “Antithrombotic agents”; **d** represents “Vasodilators used in cardiac diseases”; **e** represents “Antihypertensives”; **f** represents “Diuretics”; **g** represents “Beta blocking agents”; **h** represents “Calcium channel blockers”; **i** represents “Agents acting on the renin-angiotensin system”; **j** represents “HMG CoA reductase inhibitors”; **k** represents “Thyroid preparations”.


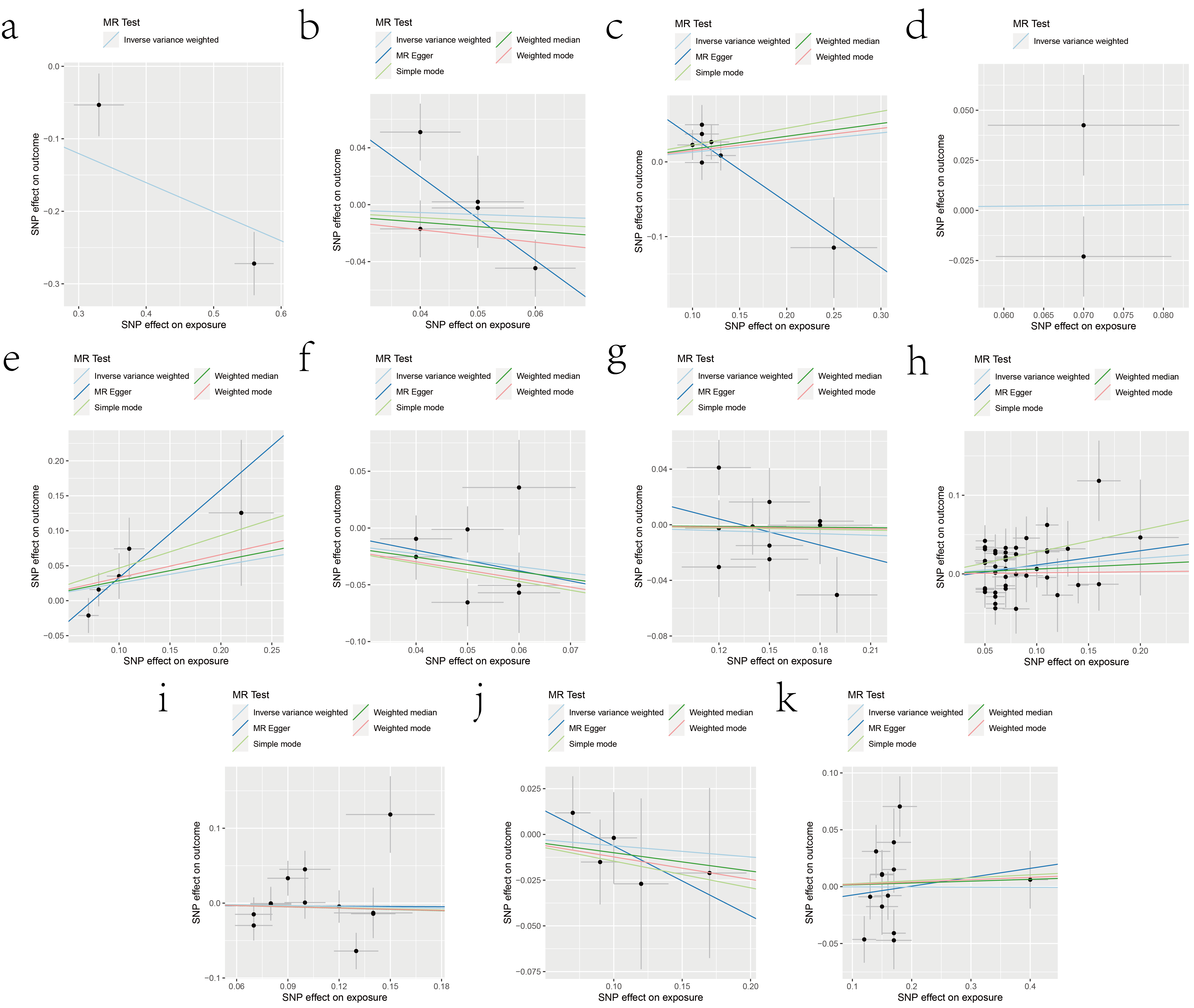


Figure 14

Note: A splicing diagram of 11 drugs and IBD from the FinnGen database.**a** represents “Immunosuppressants”; **b** represents “Anti-inflammatory and antirheumatic products, non-steroids”; **c** represents “Drugs affecting bone structure and mineralization”; **d** represents “Opioids;e represents Salicylic acid and derivatives”; **f** represents “Anilides;g represents Antimigraine preparations”; **h** represents “Adrenergics,inhalants;irepresents Glucocorticoids”; **j** represents “Antihistamines for systemic use”; **k** represents “Antiglaucoma preparations and miotics”.


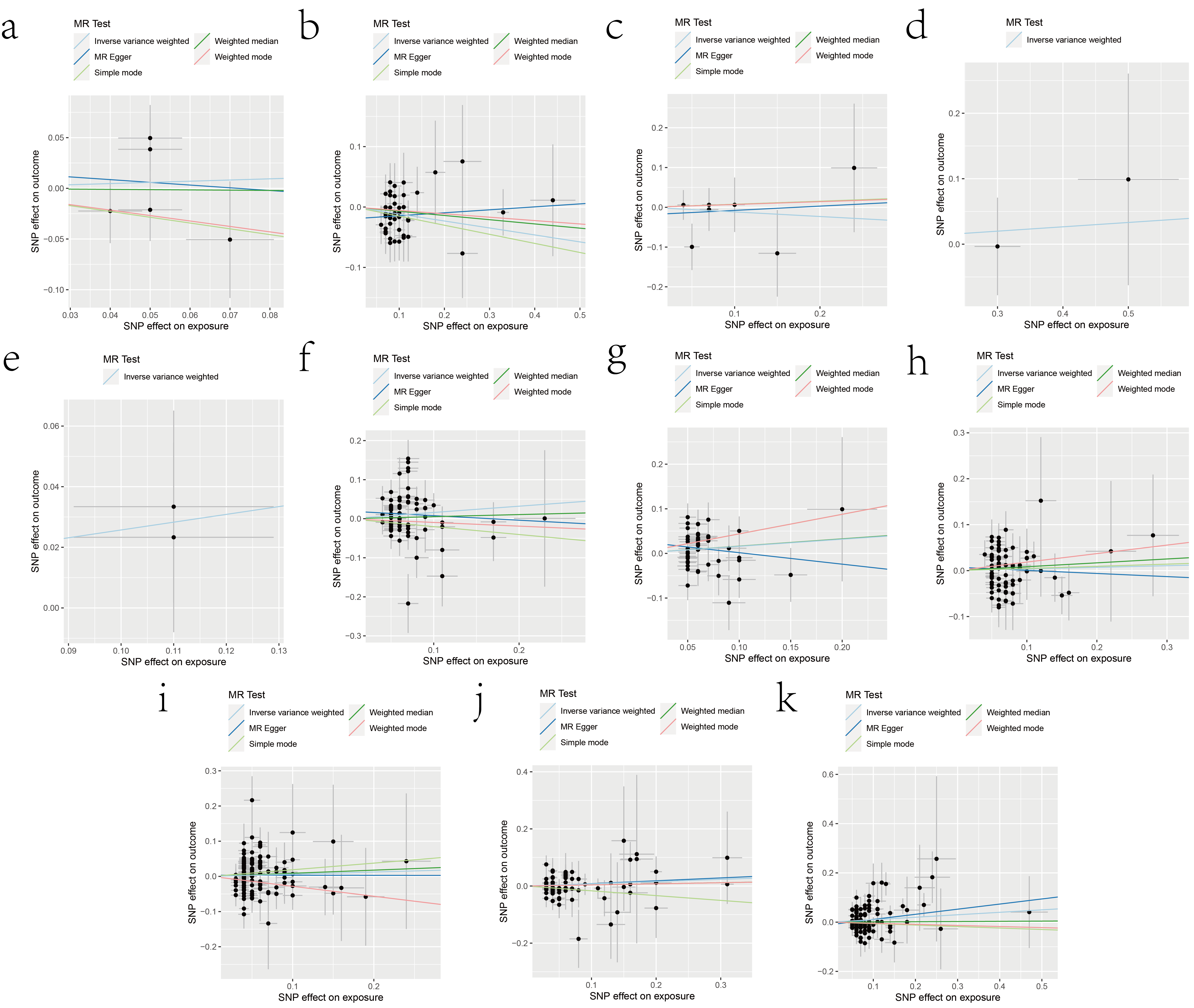


Figure 15

Note: A splicing diagram of 11 drugs and UC from the FinnGen database.**a** represents “Drugs for peptic ulcer and gastro-oesophageal reflux disease”; **b** represents “Drugs used in diabetes”; **c** represents “Antithrombotic agents”; **d** represents “Vasodilators used in cardiac diseases”; **e** represents “Antihypertensives”; **f** represents “Diuretics”; **g** represents “Beta blocking agents”; **h** represents “Calcium channel blockers”; **i** represents “Agents acting on the renin-angiotensin system”; **j** represents “HMG CoA reductase inhibitors”; **k** represents “Thyroid preparations”.


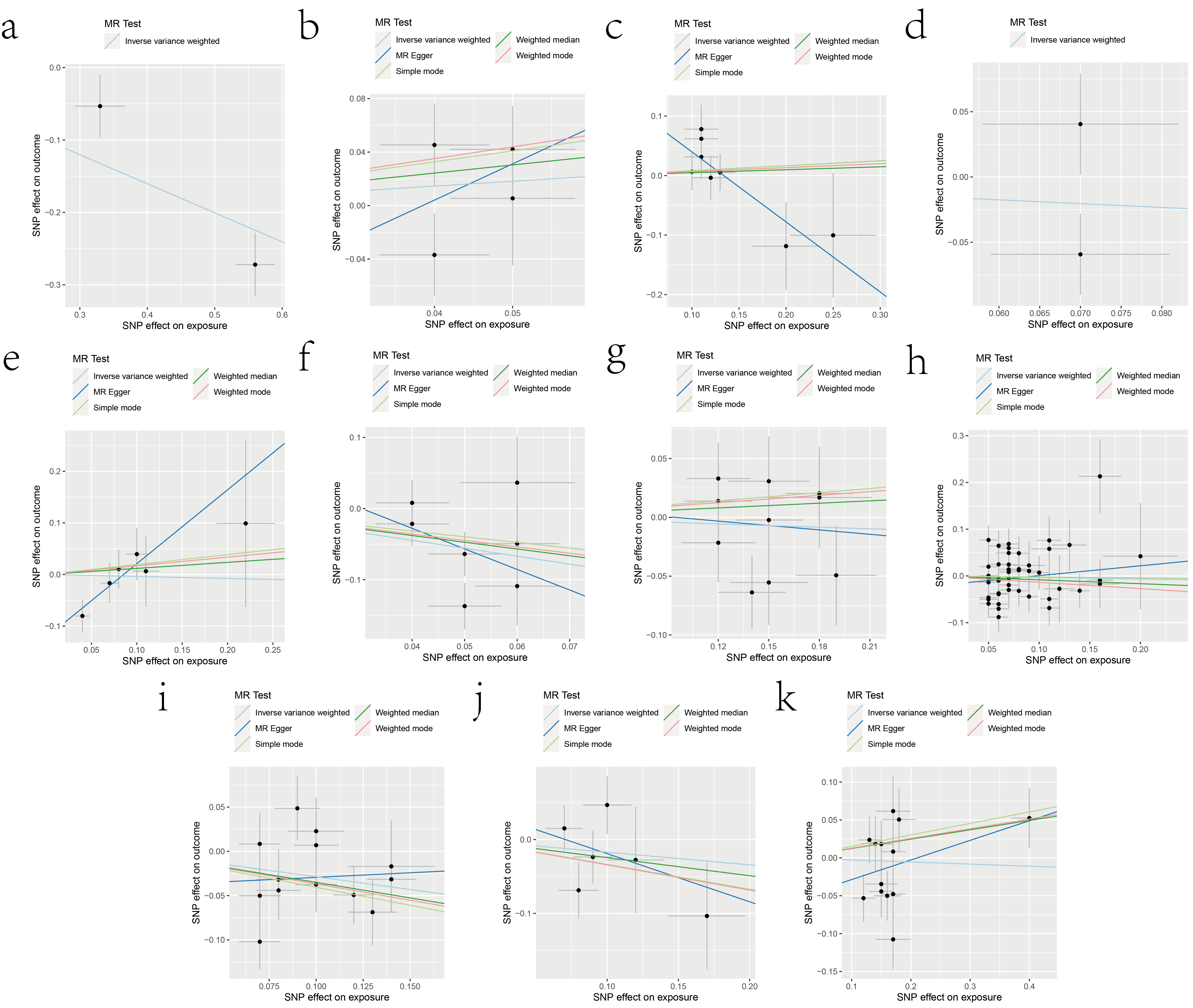


Figure 16

Note: A splicing diagram of 11 drugs and UC from the FinnGen database.**a** represents “Immunosuppressants”; **b** represents “Anti-inflammatory and antirheumatic products, non-steroids”; **c** represents “Drugs affecting bone structure and mineralization”; **d** represents “Opioids;e represents Salicylic acid and derivatives”; **f** represents “Anilides;g represents Antimigraine preparations”; **h** represents “Adrenergics,inhalants;irepresents Glucocorticoids”; **j** represents “Antihistamines for systemic use”; **k** represents “Antiglaucoma preparations and miotics”.


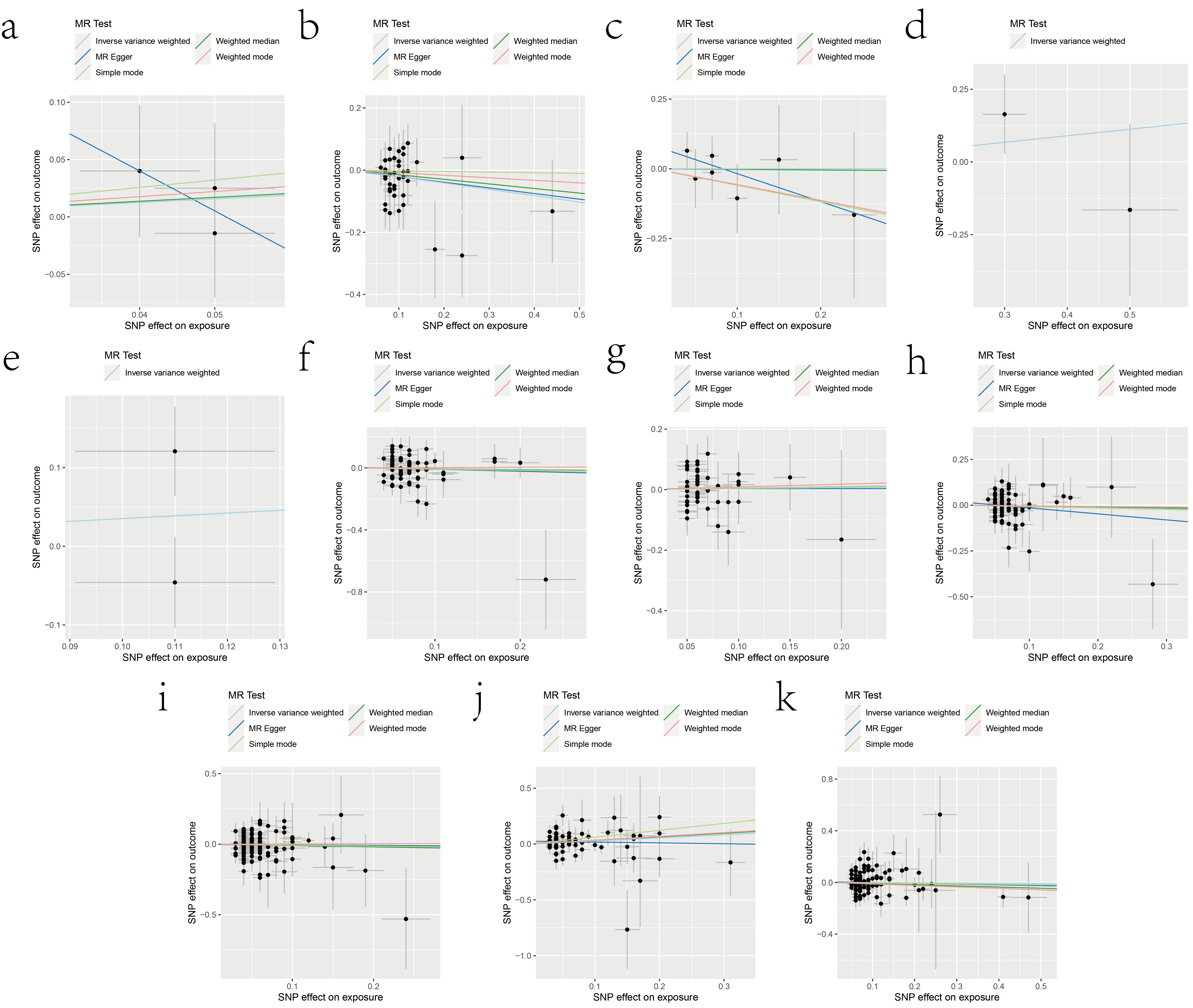
Figure 17

Note: A splicing diagram of 11 drugs and CD from the FinnGen database.**a** represents “Drugs for peptic ulcer and gastro-oesophageal reflux disease”; **b** represents “Drugs used in diabetes”; **c** represents “Antithrombotic agents”; **d** represents “Vasodilators used in cardiac diseases”; **e** represents “Antihypertensives”; **f** represents “Diuretics”; **g** represents “Beta blocking agents”; **h** represents “Calcium channel blockers”; **i** represents “Agents acting on the renin-angiotensin system”; **j** represents “HMG CoA reductase inhibitors”; **k** represents “Thyroid preparations”.


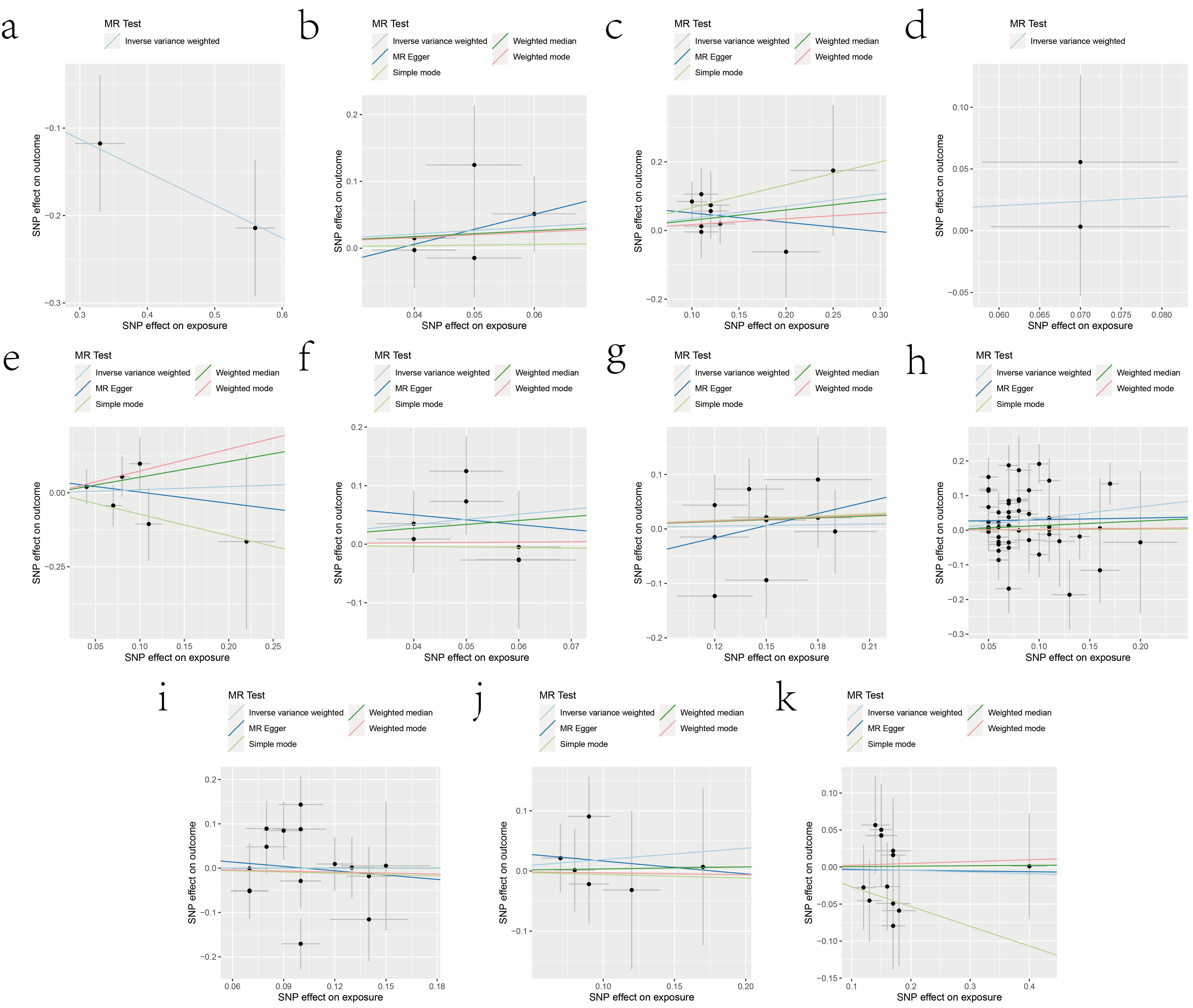


Figure 18

Note: A splicing diagram of 11 drugs and CD from the FinnGen database.**a** represents “Immunosuppressants”; **b** represents “Anti-inflammatory and antirheumatic products, non-steroids”; **c** represents “Drugs affecting bone structure and mineralization”; **d** represents “Opioids;e represents Salicylic acid and derivatives”; **f** represents “Anilides;g represents Antimigraine preparations”; **h** represents “Adrenergics,inhalants;irepresents Glucocorticoids”; **j** represents “Antihistamines for systemic use”; **k** represents “Antiglaucoma preparations and miotics”.

**The splicing diagram of scatter plots from IEU databases**


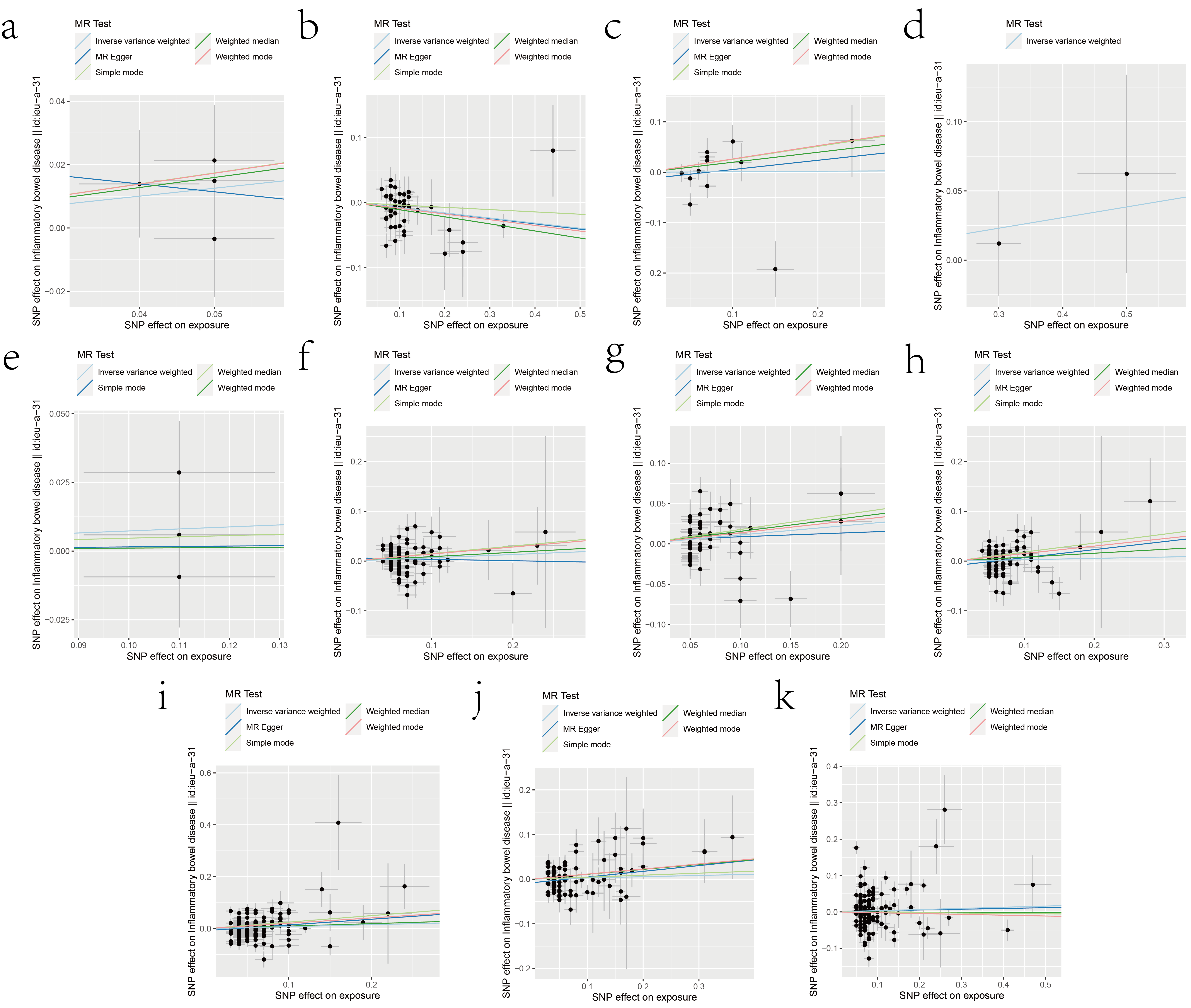


Figure 19

Note: A splicing diagram of 11 drugs and IBD from the IEU database.**a** represents “Drugs for peptic ulcer and gastro-oesophageal reflux disease”; **b** represents “Drugs used in diabetes”; **c** represents “Antithrombotic agents”; **d** represents “Vasodilators used in cardiac diseases”; **e** represents “Antihypertensives”; **f** represents “Diuretics”; **g** represents “Beta blocking agents”; **h** represents “Calcium channel blockers”; **i** represents “Agents acting on the renin-angiotensin system”; **j** represents “HMG CoA reductase inhibitors”; **k** represents “Thyroid preparations”.


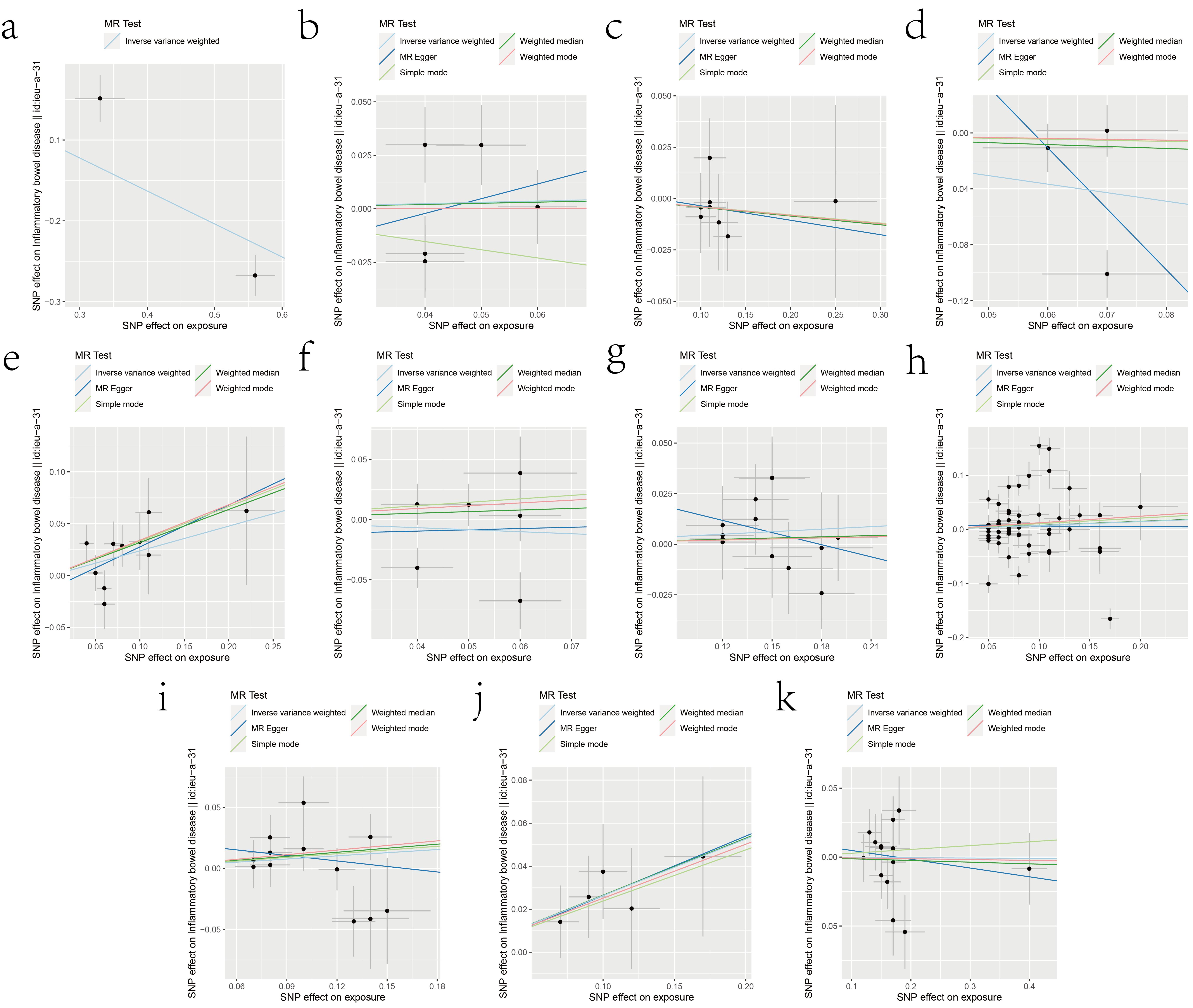


Figure 20

Note: A splicing diagram of 11 drugs and IBD from the IEU database.**a** represents “Immunosuppressants”; **b** represents “Anti-inflammatory and antirheumatic products, non-steroids”; **c** represents “Drugs affecting bone structure and mineralization”; **d** represents “Opioids;e represents Salicylic acid and derivatives”; **f** represents “Anilides;g represents Antimigraine preparations”; **h** represents “Adrenergics,inhalants;irepresents Glucocorticoids”; **j** represents “Antihistamines for systemic use”; **k** represents “Antiglaucoma preparations and miotics”.


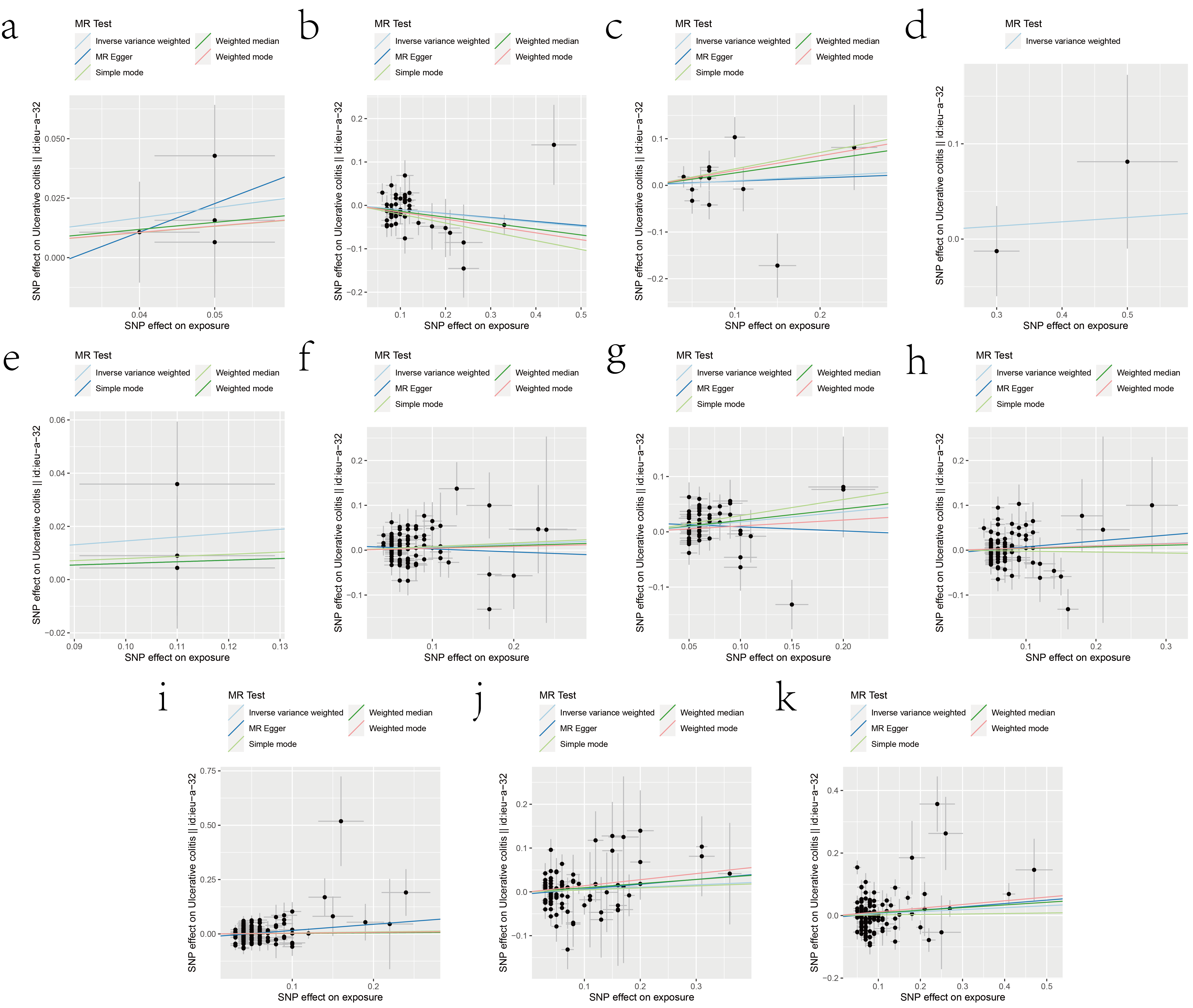


Figure 21

Note: A splicing diagram of 11 drugs and UC from the IEU database.**a** represents “Drugs for peptic ulcer and gastro-oesophageal reflux disease”; **b** represents “Drugs used in diabetes”; **c** represents “Antithrombotic agents”; **d** represents “Vasodilators used in cardiac diseases”; **e** represents “Antihypertensives”; **f** represents “Diuretics”; **g** represents “Beta blocking agents”; **h** represents “Calcium channel blockers”; **i** represents “Agents acting on the renin-angiotensin system”; **j** represents “HMG CoA reductase inhibitors”; **k** represents “Thyroid preparations”.


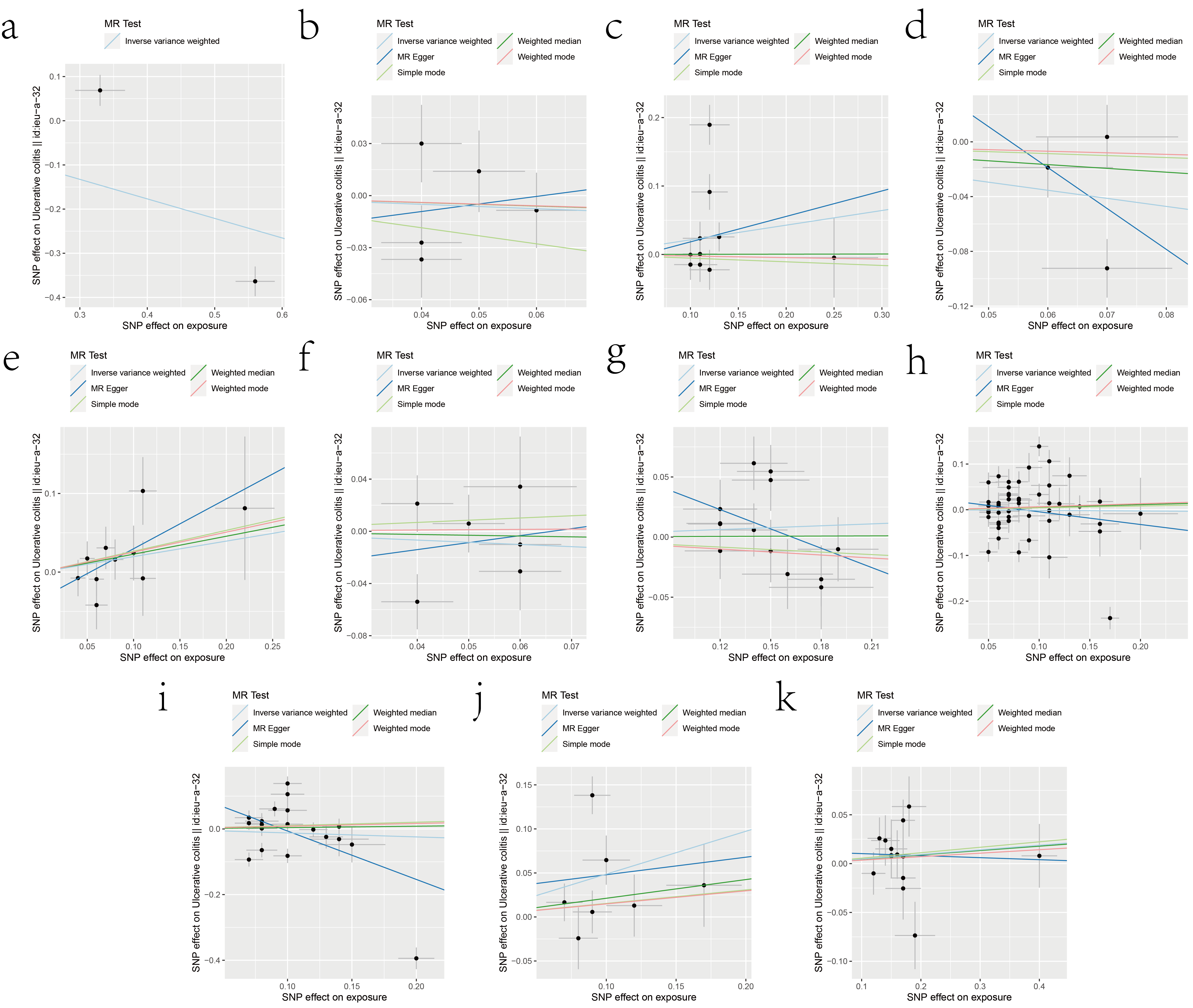


Figure 22

Note: A splicing diagram of 11 drugs and UC from the IEU database.**a** represents “Immunosuppressants”; **b** represents “Anti-inflammatory and antirheumatic products, non-steroids”; **c** represents “Drugs affecting bone structure and mineralization”; **d** represents “Opioids;e represents Salicylic acid and derivatives”; **f** represents “Anilides;g represents Antimigraine preparations”; **h** represents “Adrenergics,inhalants;irepresents Glucocorticoids”; **j** represents “Antihistamines for systemic use”; **k** represents “Antiglaucoma preparations and miotics”.


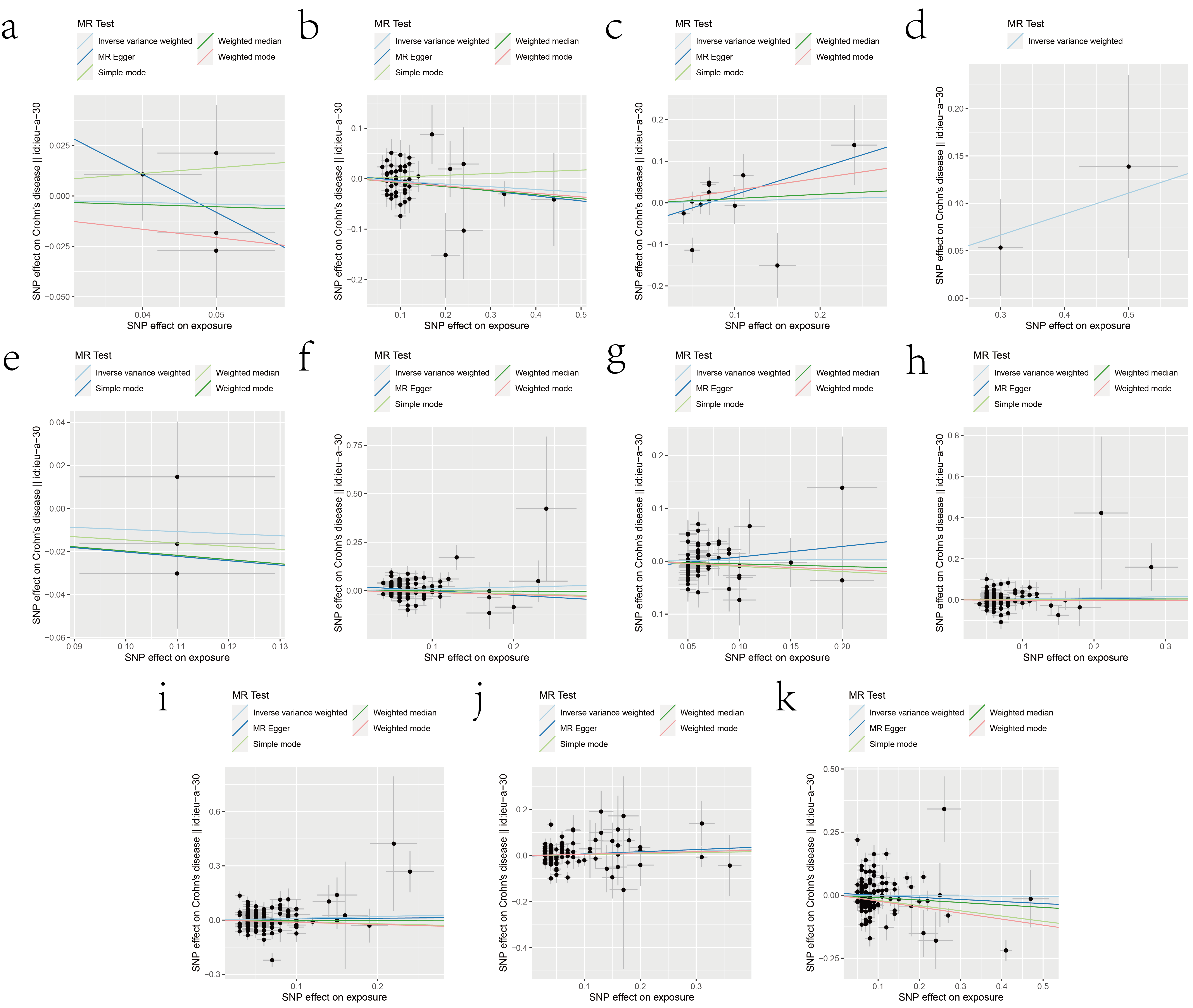


Figure 23

Note: A splicing diagram of 11 drugs and CD from the IEU database.**a** represents “Drugs for peptic ulcer and gastro-oesophageal reflux disease”; **b** represents “Drugs used in diabetes”; **c** represents “Antithrombotic agents”; **d** represents “Vasodilators used in cardiac diseases”; **e** represents “Antihypertensives”; **f** represents “Diuretics”; **g** represents “Beta blocking agents”; **h** represents “Calcium channel blockers”; **i** represents “Agents acting on the renin-angiotensin system”; **j** represents “HMG CoA reductase inhibitors”; **k** represents “Thyroid preparations”.


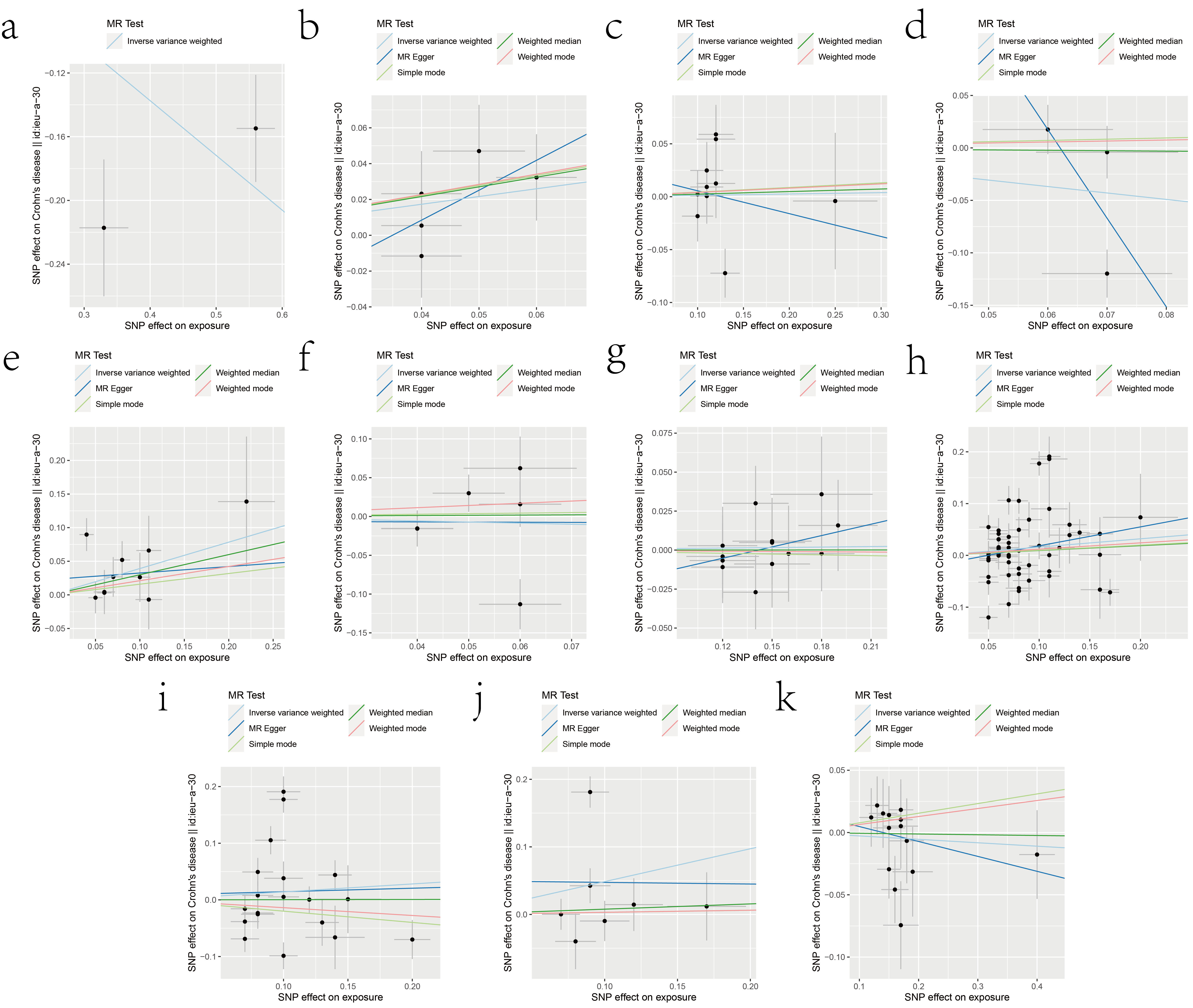
Figure 24

Note: A splicing diagram of 11 drugs and CD from the IEU database.**a** represents “Immunosuppressants”; **b** represents “Anti-inflammatory and antirheumatic products, non-steroids”; **c** represents “Drugs affecting bone structure and mineralization”; **d** represents “Opioids;e represents Salicylic acid and derivatives”; **f** represents “Anilides;g represents Antimigraine preparations”; **h** represents “Adrenergics,inhalants;irepresents Glucocorticoids”; **j** represents “Antihistamines for systemic use”; **k** represents “Antiglaucoma preparations and miotics”.
